# Establishing a Biophotonic Signature of Brain Ageing

**DOI:** 10.1111/acel.70790

**Published:** 2026-11-01

**Authors:** Mary Slayo, Loretta Vocale, Soniya Xavier, Pasindu Singhaarachchi, Kylie M. Quinn, Blanca del Rosal, Sarah J. Spencer

**Affiliations:** ^1^ School of Health and Biomedical Sciences RMIT University Melbourne Victoria Australia; ^2^ Institute of Veterinary Physiology and Biochemistry Justus Liebig, University Giessen Giessen Germany; ^3^ Department of Biochemistry, Biomedicine Discovery Institute Monash University Clayton Victoria Australia; ^4^ Department of Physics, School of Science RMIT University Melbourne Victoria Australia

**Keywords:** ageing, biomarker, fluorescence, inflammation, lipidomic, microglia

## Abstract

Ageing‐associated inflammation (“inflammaging”) is characterised by chronic microglial priming or overactivity that elevates neurodegeneration risk. Microglia also accumulate autofluorescent lipofuscin as they age–aggregates of oxidised lipids, misfolded proteins and metals that generate reactive oxygen species. Characterising age‐related changes in brain autofluorescence could provide insight into brain ageing dynamics and could identify associations with potential biomarkers in accessible tissues such as blood. We hypothesised that the ageing‐related increase in microglial autofluorescent material produces a spectral signature that makes ageing autofluorescence distinct from that in young brains. To test this, we examined autofluorescence profiles in the hippocampus and arcuate nucleus (ARC) of young (3 months) and aged (21 months) female C57BL/6 mice. We found that ageing significantly increased the volume of the autofluorescent material in microglia, including driving an abundance of extra‐large aggregates. In the ARC, larger aggregates–present only in aged brains–exhibited stronger far‐red emission than the autofluorescence seen in young brains. Fluorescence lifetime imaging analysis indicated specific ageing‐related molecular changes in brain autofluorescence. Lipidomic analysis from the plasma of these mice indicated 22 lipids that were differentially concentrated with age and one (sphingomyelin 32:2) that was significantly associated with the ageing brain spectral profile after controlling for multiple comparisons and validating in a separate cohort. These findings demonstrate that autofluorescent material aggregates with age in microglia and that brain autofluorescence undergoes a spectral red‐shift associated with ageing and lipid alterations. Brain autofluorescence may thus reflect brain ageing with potential translational relevance for non‐invasive monitoring.

## Graphical abstract

If we excite autofluorescence from brains with a 488 nm laser, young brain autofluorescence shows blue‐shifted spectra; aged brain autofluorescence shows red‐shifted spectra. This spectral shift is predicted by plasma SM32:2 levels. These signals allow unbiased distinction of an ageing from a young brain.

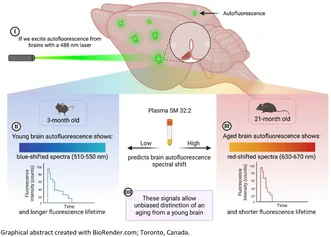

## 1 Introduction

Even healthy ageing is accompanied by gradual development of chronic low‐grade inflammation known as “inflammaging”, which is characterised by elevated inflammatory biomarkers (Franceschi et al. 2018). In the brain, chronic low‐grade activation of the immune system elicits the production of cytokines that amplify the pro‐inflammatory response and degrade adjacent neurons at the expense of healthy tissue (Franceschi and Campisi 2014). Age‐related factors that contribute to inflammaging include obesity, a dysregulated immune response, oxidative stress, dysfunctional mitochondria, and cellular senescence (Ferrucci and Fabbri 2018). This development not only increases the risk of ageing‐related and inflammatory‐related neurodegenerative diseases but also accelerates the ageing process and primes the immune system to induce greater inflammatory responses to subsequent stimuli (Franceschi et al. 2018). However, early physiological changes that distinguish normative ageing and heightened neurodegenerative vulnerability remain incompletely defined.

This ageing response is contributed to by microglia, the innate immune cells within the central nervous system (CNS) that maintain tissue homeostasis through repair (Sominsky et al. 2018). In the presence of injury, infection or damage, microglia adopt a functionally and morphologically responsive phenotype by increasing phagocytosis and releasing pro‐inflammatory mediators, chemokines and apoptotic factors. Specific ligands attach to microglia's membrane‐bound toll‐like receptors (TLRs) to trigger the inflammatory process, activating signalling molecules to upregulate phagocytosis, secrete cytokines, and increase expression of co‐stimulatory molecules (Lyman et al. 2014). However, chronic low‐grade release of pro‐inflammatory cytokines, including tumour necrosis factor (TNF) and members of the interleukin (IL) family, causes dystrophy in nearby neurons (Lyman et al. 2014). Chronic overactivity of this response is suggested to increase the risk of and exacerbate damage in ageing‐associated neurodegenerative diseases such as Alzheimer's disease (Hickman et al. 2018). With ageing, microglia adopt a “primed” phenotype characterised by exaggerated and uncontrolled inflammatory responses to immune stimuli (Perry and Holmes 2014). Primed microglia become less ramified, more phagocytic (Spencer et al. 2019), and overproduce cytokines in response to immune stimuli (Frank et al. 2010). Since microglia play a pivotal role in maintaining neuronal health, their hyperactivity in ageing may disrupt synaptic plasticity and lead to memory loss (Chapman et al. 2012). This makes microglia priming a likely contributor to cognitive deficits and increased risk of neurodegenerative diseases with age. Although transcriptional and morphological alterations have been described extensively, it remains unclear whether microglial ageing can be delineated by a quantifiable biophysical phenotype.

Recent work has suggested that the microglial response in healthy ageing can be characterised by the accumulation of autofluorescent material that displays spectrally broad excitation and emission profiles (Burns et al. 2020). This material has been classified under the umbrella term “lipofuscin” that includes mixed aggregates of fluorescent oxidised lipids, misfolded proteins and metals that produce high levels of reactive oxygen species (Moreno‐García et al. 2018). Lipofuscin has broad excitation and emission spectra with an optimal excitation range between 320 and 480 nm, and emission between 460 and 630 nm (Moreno‐García et al. 2018). Microglia containing autofluorescent material in flow cytometry analyses have been shown to have increased endolysosomal, autophagic, and metabolic activity (Burns et al. 2020). They demonstrate increased oxidative phosphorylation and fatty acid β‐oxidation as well as dysfunction in mitochondrial respiration relative to those that do not show autofluorescence with this technique (Burns et al. 2020). Our group has recently demonstrated with fluorescence microscopy that all brain microglia contain at least some autofluorescent material and that its size and volume changes acutely (within 13 h) in accordance with an acute neuroinflammatory challenge (Slayo et al. 2026). These observations raise the possibility that autofluorescent aggregates reflect dynamic metabolic and inflammatory states rather than, or in addition to, being passive by‐products of cellular ageing. However, while microglial autofluorescence is increasingly recognised as a feature of ageing, it is unknown whether it reflects a structurally and spectrally distinct ageing phenotype that can be quantitatively defined.

It also remains unclear whether such CNS alterations are accompanied by peripheral biochemical changes detectable in the circulation. Lipid profile analysis is a clinically relevant method that may be better able to capture early disease changes in metabolism, energy storage, and cell structure than proteomics. Lipidomic analyses have revealed complex relationships between distinct classes of lipids and DNA methylation as a measure of biological ageing in humans (Liu et al. 2023). These findings suggest the possibility of a link between ageing‐related changes in cell dynamics reflected in endogenous fluorophores within brain microglia and systemic lipid composition. We therefore hypothesised that ageing is associated with the accumulation of autofluorescent material inside microglia and that this is reflected in alterations to peripheral lipids. We assessed the hippocampus due to our interest in neurodegenerative diseases and the arcuate nucleus of the hypothalamus (ARC) because of our interest in inflammation. Here we show that there is a greater volume of autofluorescent material in the mouse brain with ageing and that this occurs particularly in microglia. We use photoluminescence (PL) spectroscopy and fluorescence lifetime imaging microscopy (FLIM) to reveal that this material has a relatively red‐shifted emission peak spectra and shorter autofluorescence lifetime in the aged mouse. This ageing‐related increase in autofluorescent material is associated with changes in genes related to inflammation (*Aif1, Tlr4, Itgam, Mhc‐II*). Finally, these ageing‐related spectral changes in brain autofluorescence are associated with lipid changes in the plasma across independent data sets. Together, these data support the idea that ageing‐related shifts in the photophysical behaviour of endogenous fluorophores are coupled to systemic biochemical alterations, providing a quantitative basis for tracking early physiological changes associated with neurodegenerative risk.

## 2 Materials and Methods

### 2.1 Animals

All experiments were conducted in accordance with the Australian Code of Practice for the Care and Use of Animals for Scientific Purposes. Tissues were harvested from naïve animals being used in other studies of mouse ageing, with approval from the RMIT University Animal Ethics Committee (AEC #22406, #24986 and #23208). Naïve female C57BL/6 mice (Ozgene, Perth, WA, Australia) of 3 (young) or 21–22 (aged) months of age were used. Mice were kept under standard laboratory housing conditions in the RMIT University Animal Facility, with a 12‐h light cycle (lights gradually on from 6 am and gradually off from 6 pm); temperature, 18°C–24°C; humidity, 40%–70%. Each cage was supplied with plastic tunnels and shredded paper as enrichment. The mice were given *ad libitum* access to water and standard rodent chow diet (Specialty Feeds, WA, Australia).

### 2.2 Tissue Collection

Mice were humanely killed at 3 or 21–22 months old using isoflurane inhalation followed by CO_2_. All mice were transcardially perfused with 1× phosphate buffered saline (PBS: 4°C, pH 7.4). Brains were collected and hemisected along the sagittal fissure. From one hemisphere, the hippocampus was dissected, snap‐frozen in liquid nitrogen, and stored at −80°C until use in quantitative real‐time PCR (qrt‐PCR). The other hemisphere was immersion‐fixed overnight at 4°C in 4% paraformaldehyde in PBS for later immunohistochemistry. After post‐fixation, the half brains were transferred to 20% sucrose in PBS (4°C). Brains were cut into 30 μm coronal sections using a cryostat into a one‐in‐five series and stored in 0.02% sodium azide in PBS at 4°C until use.

### 2.3 Quantitative Rt‐PCR

To assess ageing‐related changes in hippocampal gene expression, we performed qrt‐PCR as previously described (de Luca et al. 2019). Briefly, we extracted RNA from the hippocampus using QIAzol reagents and RNeasy Mini Kits (Qiagen, Valencia, CA, USA). RNA concentrations and quality were assessed using a spectrophotometer (NanoDrop 2000/2000c; Thermo Fisher Scientific, Wilmington, DE, USA). cDNA was synthesised using the QuantiTect Reverse Transcription kits (Qiagen) as per the manufacturer's instructions. qRT‐PCR was then performed using Taqman Gene Expression Assays (Thermo Fisher Scientific) on a QuantStudio 7 Flex instrument (Applied Biosystems, Carlsbad, California, US). We assessed changes in the expression of markers involved in inflammation (*Aif1, Tlr4, Nf‐κB, Mtor, Itgam*, and *Sirt1*), lipid pathways (*Abcg1, Acat1, Hexb, Ptdss1, Ch25h, Lpl*) and genes associated with Alzheimer's disease (*App, Bace2, Sorl1, Mapt, Abca7*). Primer details are shown in Supplementary Table 1. We normalised a relative quantitative measure of the target gene expression with the geometric mean of the two endogenous controls, *Gapdh* and *Hprt*. mRNA expression was analysed using the equation 2^−ΔΔC(t)^, where C(t) is the threshold cycle at which fluorescence is first detected significantly above background.

### 2.4 Immunolabelling

To verify our mice were displaying an expected ageing profile, we immunolabelled sections through the hippocampus for doublecortin (DCX), a marker for immature neurons, as described previously (Xavier et al. 2021). Young and aged brain sections were run on the same immunohistochemistry plate to avoid variability between runs. The primary goat anti‐DCX antibody (1:500; Santa Cruz Biotechnology Inc., Dallas, TA, USA) was diluted in blocking buffer solution and sections were incubated overnight at 4°C. Following the incubation period, sections were washed 3 × 10 min in 1 × PBS to remove unbound primary antibody and were then incubated with the secondary biotinylated anti‐goat antibody (1:500; Vector Laboratories, Newark, CA, USA) in blocking buffer for 2 h at room temperature. Brain sections were mounted onto slides, air‐dried overnight, dehydrated in a series of alcohols, cleared in histolene and cover slipped using dibutylphthalate polystyrene xylene (DPX) mounting medium.

Counts of DCX‐positive cells were completed using an Olympus upright microscope (Olympus BX41; Olympus, Melbourne, Vic, Au) with a ×40 objective lens and an Olympus DP72 digital camera (Olympus) with LabSens image capture software v1.6 (Olympus). Images were taken at a 4140 × 3096 pixel density. Hippocampal brain sections were identified according to the Allen Mouse Brain Atlas (2004) and we examined three sections across the dentate gyrus (DG) between 1.65 and 2.35 caudal to the bregma.

To co‐localise and assess the level of autofluorescence within different brain cells in ageing, we used standard immunofluorescent labelling techniques. Coronal sections across the hippocampus were immunolabelled for NeuN and across the hippocampus and ARC for ionised calcium binding adaptor molecule 1 (IBA1), a marker for microglia. Briefly, tissue from a one‐in‐five series was washed 3 × 10 min in 1 × PBS then blocked for 1 h with BSA, 0.3% Triton X‐100 in 1 × PBS. The sections were separately incubated in the primary antibody (NeuN: rabbit, 1:5000, Abcam, Cambridge, England, UK; IBA‐1: rabbit, 1:1000, Wako Pure Chemical Industries, Osaka, Japan) for 16–18 h at room temperature. The sections were washed 3 × 10 min in 1 × PBS, then incubated in secondary antibody (Alexa Fluor647 goat anti‐rabbit) in a 1:500 dilution in 1% BSA for 2 h at room temperature. Sections were washed again with 1 × PBS and mounted onto Superfrost slides, air dried and then cover slipped with Dako fluorescence mounting medium (Agilent, Santa Clara, CA, USA).

To assess the autofluorescence signal within the brain, autofluorescence images were captured using 488 nm excitation and a 500–600 nm emission bandpass, excitation and emission wavelengths we have previously categorised as optimal for measuring brain autofluorescence (Slayo et al. 2026). IBA1‐ and NeuN‐ (Alexa 647) labelled images were captured using 640 nm excitation and a 650–750 nm emission bandpass. All z‐stack images were taken at ×100 magnification for each animal using an Olympus FV3000 confocal microscope.

### 2.5 Morphological and Autofluorescence Analysis

To assess if ageing was associated with a change in microglial complexity, Imaris version 10 (Bitplane, Zurich, Switzerland) with MATLAB R2017b version 9.8 was used to perform Sholl analysis. The hilus region of the DG, CA1 and CA3 hippocampus, as well as the ARC, were analysed. Two to three microglia were used from each of four images from sections 120 μm apart to obtain a total analysis of at least seven representative cells per region, maintaining a standardised sampling strategy for all animals. Microglia were excluded if their processes extended beyond the image frame and were otherwise selected randomly by the software. The mean number of intersections at each 1 μm interval extending from the periphery of the cell body was calculated for each animal. Data were exported to Microsoft Excel and GraphPad Prism software (version 9.0.1) for collation and statistical analysis.

Confocal Z‐stacks of each brain region were used to analyse the volume and intensity of autofluorescent material, both across the entire region and specifically within microglia, using a custom‐made macro in Fiji/ImageJ (see Supplementary Methods for macro code). Our macro used CLIJ2 for GPU‐accelerated image processing (Haase et al. 2020), MorphoLibJ for morphological and volume‐based filters (Legland et al. 2016), and Biovoxxel 3D Box for object analysis (Brocher 2025). Briefly, the autofluorescence and IBA1 channels in the confocal Z‐stacks were first smoothed and then processed independently. Autofluorescent granules were identified from the autofluorescence channel using the MaxEntropy thresholding method, while a series of morphological filters were applied to the IBA1 channel to isolate microglia soma. Label maps generated from both images were used to analyse the overlap between autofluorescence and IBA1 signal using Biovoxxel's object inspector. The output from the macro was visually inspected and manually revised and any cells meeting the exclusion criteria were removed from the analysis. Any cells missing from the soma mask were analysed by manually selecting regions of interest for each microglia soma. The mean volume of autofluorescence was obtained for each animal, and the autofluorescence data were also binned to obtain proportions of small (0.1–10 μm^3^), medium (10.01–20 μm^3^), large (20.01–50 μm^3^) and extra‐large (50.01–100 μm^3^) aggregates.

### 2.6 Autofluorescence Emission Spectra Measurements

To assess if ageing was associated with spectral changes in brain autofluorescence, emission spectra of autofluorescent aggregates were recorded using a spectrometer (Princeton Instruments Isoplane 320, Trenton, New Jersey, USA) equipped with a 150 lines/mm diffraction grating and coupled to an intensified CCD camera (PI‐MAX 4 1024, Trenton, New Jersey, USA). This was connected to the confocal microscope through a multimode optical fibre. Unstained tissue was used to avoid spectral interference from the secondary antibody fluorescence. Spectra were recorded in the 490–750 nm emission range under 488 nm optical excitation. Acquisition was averaged across 40 frames. The autofluorescence emission spectrum was recorded for three autofluorescent aggregations per animal in the young. In every case these were small (0.5–1.5 μm diameter) since no larger ones were evident. For aged, we measured three aggregations of a small size (defined as 0.5–1.5 μm diameter to match those found in the young and because aged tissue did not contain any smaller autofluorescence) and three large (defined as 1.5–10 μm diameter) per animal. Spectra were analysed using OriginLab (Origin 2024b, OriginLab Corporation, Northampton, Massachusetts, USA). A mean spectrum was obtained for each animal (and size category for aged) by averaging the spectra from individual aggregates after pre‐processing and normalisation. Briefly, pre‐processing consisted of spike removal of cosmic rays and subtraction of a constant baseline, calculated from the average intensity between 489 and 496 nm, followed by smoothing with a 63‐point Savitzky Golay filter. To assess any changes in the peak emission wavelength, we calculated the ratio of the integrated intensities across two wavelength bands (I(1), 515–555 nm; I(2), 630–670 nm) for each mean spectrum. These bands were selected for flanking the peak maxima and demonstrating low inter‐animal variability within age groups. To resolve overlapping peaks within these spectra, the second derivative was calculated for each mean spectrum using a Savitzky–Golay filter (55 points of window, 2nd order polynomial). Mean spectra and second derivative curves for autofluorescent aggregates of the same size were averaged to obtain one spectral profile for the young animals and two for the aged (a small‐autofluorescence aggregation spectrum and a large‐autofluorescence aggregation spectrum). Mean intensity ratios were then calculated for each of the small and large aggregation sizes in each age group. Peaks in the second derivative curves were analysed for all animals, and the peaks that were present in > 90% of the animals were selected for comparison between groups.

### 2.7 Fluorescence Lifetime Imaging

To assess if ageing was associated with fluorescence lifetime changes in the autofluorescence signals, we used a PicoQuant FLIM system integrated with an Olympus FV3000 confocal microscope (Supplementary Figure 1). A pulsed 485 nm laser (LDH‐D‐C‐485; PicoQuant GmbH, Berlin, Germany) operating at 13.33 MHz (average power 10.2 μW) was used for optical excitation. To collect lifetime data for the full spectrum, the fluorescence emission was filtered through a 488 nm longpass filter (Semrock, BLP01‐488R) and collected using a hybrid photomultiplier detector (PicoQuant PMA Hybrid 40) connected to a multichannel time‐correlated single photon counting module (PicoQuant MultiHarp 150; Supplementary Figure 1) with a temporal resolution of 80 ps. To collect lifetime data across two different spectral bands, a fluorescence filter cube with a 635 nm longpass dichroic beamsplitter (Semrock, Di02‐R635‐25 × 36) was used to split the fluorescence into two channels, each equipped with an additional spectral filter, a 550/50 nm bandpass filter (Edmund Optics, Barrington, New Jersey, USA) and a 664 nm longpass filter (Semrock, West Henrietta, New York, USA). PicoQuant SymphoTime 64 software was used for image acquisition and lifetime analyses. Images were acquired at 10 μs/pixel and a resolution of 0.025 μm/pixel with a defined region‐of‐interest around the autofluorescent material only. Lifetime decay curves were obtained by scanning each area for 1000 cycles (young) or 500 cycles (aged) to obtain similar photon counts for analysis since the young autofluorescent aggregates were dimmer.

### 2.8 Plasma Collection and Lipid Extraction

To determine whether we could identify peripheral changes that reflect brain autofluorescence shifts with ageing, we conducted lipidomic analysis in plasma from the same animals. Peripheral blood up to 1.5 mL was collected by cardiac puncture using a syringe pre‐coated with 0.5 M EDTA (ThermoFisher Scientific, Waltham, Massachusetts, USA) attached to a 23 G needle, transferred to an EDTA‐coated Eppendorf microtube and mixed by gentle inversion of the tubes. Up to 400 μL of plasma was separated from whole blood by centrifugation at 1500 × *g* for 15 min at 4°C and stored at −80°C until use. Plasma lipids were extracted using a modified butanol: methanol protocol. Briefly, 10 μL of plasma was mixed with 100 μL of chilled butanol: methanol (1:1, v/v) containing internal standards at a final concentration of 10 mg/L. The internal standard mixture comprised phosphatidylcholine (PC) 19:0/19:0, phosphatidylglycerol (PG) 17:0/17:0, and the deuterated triacylglycerol TG‐d5 (Avanti Polar Lipids, Alabama, USA). This extraction solution was used for all samples to monitor sample processing and instrument performance. Samples were vortexed for 30 s, followed by thermomixing at 950 rpm for 5 min at 10°C using an Eppendorf Thermomixer (Eppendorf South Pacific Pty Ltd., Macquarie Park, Australia). After centrifugation at 15,000 rpm for 5 min, the supernatant containing extracted lipids was transferred into high‐performance liquid chromatography (HPLC) vials for analysis.

To assess instrument stability, pooled biological quality control (PBQC) samples were prepared by combining 10 μL aliquots from each sample extract into a single vial. Samples were injected in random order, with PBQC injections (2 μL) performed after every five study samples. Lipid profiling was conducted using liquid chromatography‐triple quadrupole mass spectrometry (LC‐QQQ‐MS), and features exhibiting a PBQC coefficient of variation (CV) greater than 30% were excluded from downstream analysis.

### 2.9 Liquid Chromatography‐Triple Quadrupole Mass Spectrometry (LC‐QQQ‐MS)

Targeted lipidomic analysis was performed by Metabolomics Australia (Bio21, Parkville, Victoria, Australia) using an Agilent 1290 liquid chromatography (LC) system and Triple Quadrupole 6490 mass spectrometer (MS, Agilent Technologies Australia, Mulgrave, Victoria, Australia) following established protocols (Huynh et al. 2018). Briefly, plasma (2 μL) was separated using a Zorbax Eclipse Plus C‐18 column (2.1 × 100 mm, 1.8 μm; Agilent Technologies Australia) at 45°C at a rate of 0.4 mL/min with a mobile phase solvent system of A (50% water, 30% acetonitrile, 20% isopropanol) and B (1% water, 9% acetonitrile, 90% isopropanol). Both solvents contained 10 mM ammonium formate. A stepped elution gradient was used as previously described (Huynh et al. 2018) with a total run time of 15 mins. The lipids were detected in positive ionisation mode with dynamic scheduled multiple reaction monitoring (MRM). Quantitation was based on relative changes in peak areas. Data processing was performed using Agilent's Mass Hunter Quantitative Analysis software (Agilent Technologies Australia). Lipids were named according to the nomenclature described in LIPID MAPS (Liebisch et al. 2013).

### 2.10 Lipidomic Data Preprocessing and Analysis

A curated list of 426 metabolites (from the targeted 510 metabolites in the Mass Hunter Database/Mass Hunter Quantitative Analysis) was obtained and data imported into MetaboAnalyst 5.0 for initial quality control checks and analysis by Metabolomics Australia (Bio21 Institute, Melbourne, Victoria, Australia). This curated dataset was subject to further downstream statistical analysis as follows.

Peak area data for each lipid compound was uploaded to MetaboAnalyst (https://www.metaboanalyst.ca/) for analysis. In the software, data were filtered to remove lipids with low repeatability (relative standard deviation > 20%), low variance (10% of interquartile range) and low abundance (10% of mean intensity value). After filtering, 337 lipids remained. Data were processed using sum normalisation and log_10_ transformation. Principal component analysis (PCA) and supervised orthogonal partial least squares discriminant analysis (OPLS‐DA) were performed to identify outliers. No outliers were identified so all data were retained. A fold‐change threshold of 1.5 (log2FC = ±0.585) and false discovery rate (FDR) of 0.1 (−log_10_ *p* = 1.0) were set to determine differences in lipid metabolites between age groups. Hierarchical clustering of the significantly changed lipids was performed utilising Euclidean distance and Ward's linkage clustering and visualised as a heatmap. We also performed simple linear regressions between the age‐related lipids and brain autofluorescence spectra with a Bonferroni corrected cut‐off of *p* < 0.0023. The *R* and *R* ^2^ values of the significantly associated lipids with Bonferroni correction are presented to reflect the variance of spectra explained by changes in the lipids.

### 2.11 Spectral and Lipidomic Analysis Validation

To test whether these plasma changes were robustly associated with brain autofluorescence changes with ageing across cohorts, we replicated the spectral ratio and lipidomic analyses in a separate validation cohort of ageing mice. For this validation cohort, plasma collection, lipid extraction and LC‐QQQ‐MS were performed as described above in *n* = 6 young and *n* = 7 aged mice. In the validation cohort, a curated list of 422 metabolites was obtained and data imported into MetaboAnalyst 5.0 following the same process as described including filtering, normalisation to sum, and log_10_ transformation. We note that one of the aged animals was identified post‐mortem to have a brain tumour (that did not make up part of the sample) and was also identified as an outlier using PCA analysis. For the main analyses presented throughout, the animal was excluded since this represents a qualitatively different pathological state from ageing‐related neurodegeneration. However, we aimed to identify a measure that can reflect a broad range of disease stages across ageing, not just healthy ageing. Therefore, we have performed separate spectral and regression analyses with this animal included in the Supplementary Materials for transparency and the readers' interest.

### 2.12 Data Analysis

All analyses except the lipidomic were conducted by an experimenter blinded to any treatment conditions. GraphPad Prism software (version 9.0.1) was used to perform all statistical testing and graphing except where otherwise indicated. All analyses, except for the lipidomic dataset (which was analysed as described above), were preceded by non‐iterative Grubbs' test for outliers and the Shapiro–Wilk test for normality.

Student's unpaired *t*‐tests were used to compare the two age groups for: number of DCX‐, NeuN‐ and IBA1‐positive cells, DCX categorisation, total volume of autofluorescence, fold change in gene expression, and fluorescence lifetime for each emission band. For the microglial Sholl and autofluorescence size bin categorisation, Mauchly's test was used to determine sphericity, and the data were analysed in bins using one‐way repeated measures analysis of variance (ANOVA) with Tukey's *post hoc* comparisons in SPSS 29. For the spectral data, differences in the normalised spectral intensity at each wavelength peak were analysed using a mixed model ANOVA, while the mean intensity ratios were analysed using a one‐way ANOVA. Both were followed with Tukey's *post hoc* analyses where significant interactions or main effects, respectively, were found. Simple linear regressions of spectra ratio and our target plasma lipids were performed in SPSS 29 and graphed in GraphPad Prism. Data are presented as mean ± SEM and statistical significance was assumed when *p* ≤ 0.05.

## 3 Results

### 3.1 Ageing‐Related Changes in Brain Cell Populations

Here, to establish that our ageing cohort were displaying expected ageing‐related brain changes in neurogenesis, we examined the ageing‐related differences in neuron numbers and maturity. To do this, we assessed the number of cells positive for DCX (Figure 1A–D) and NeuN (Figure 1E). Ageing significantly reduced the total number of DCX‐positive neurons in the DG of the hippocampus (*t*_(8)_ = 18.19, *p* < 0.0001; Figure 1A). This was the case for all maturation stages of the neurons (proliferative: *t*_(8)_ = 9.16, *p* < 0.001; intermediate: *t*_(8)_ = 6.32, *p* < 0.001; and post‐mitotic: *t*_(8)_ = 15.3, *p* < 0.001; Figure 1B–D,F,G). However, there was no difference between the ages in the total number of NeuN‐positive neurons in the same region (Figure 1E,H,I).

**FIGURE 1:**
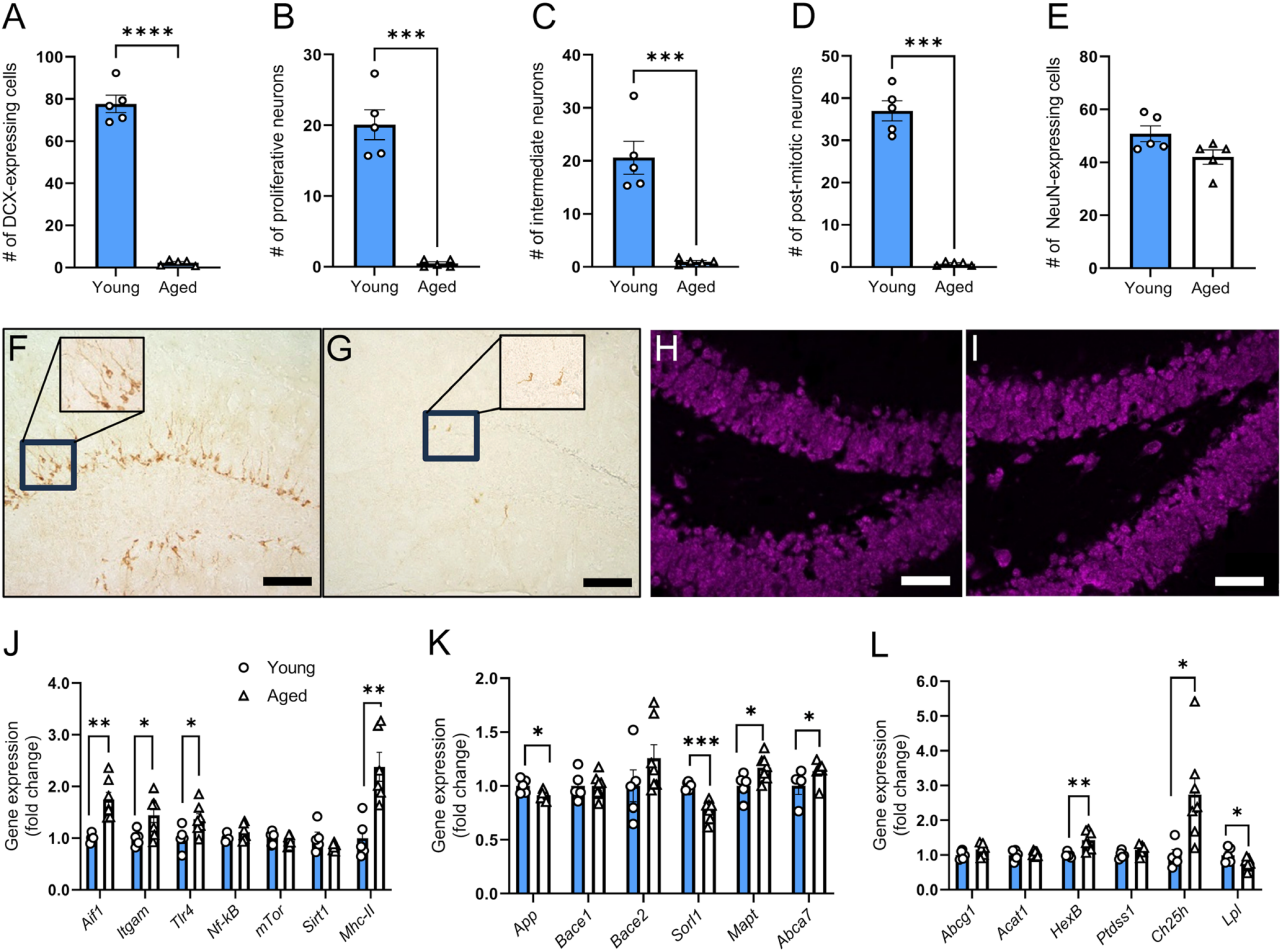
Ageing leads to a reduction in the number of new hippocampal neurons and to changes in hippocampal gene expression in otherwise healthy mice. (A) Total number of doublecortin (DCX)‐positive cells in the hippocampal dentate gyrus (DG) of young and aged mice. (B–D) Number of (B) proliferative, (C) intermediate and (D) post‐mitotic DCX‐positive neurons. (E) Total number of neuronal nuclear protein (NeuN)‐positive hippocampal DG cells. (F, G) Representative images of DCX‐positive cells in (F) young and (G) aged mice with an inset in each image. Scale bars = 100 μm. (H, I) Representative images of NeuN‐positive cells in (H) young and (I) aged mice. Scale bars = 45 μm. (J–L) Age‐related changes in expression of genes relating to (J) inflammation, (K) Alzheimer's disease and (L) lipid pathways. *p < 0.05; **p < 0.01; ***p < 0.001; ****p < 0.0001. Data are mean ± SEM. *n* = 5–7 per group.

To characterise the age‐related changes in ageing‐associated gene expression in these mice, we assessed genes relating to inflammation and lipid transport, as well as genes known to be altered in Alzheimer's disease. With age, there was a significant increase in the expression of inflammation‐related *Itgam* (*t*_(10)_ = 2.45, *p =* 0.034), *Tlr4* (*t*_(10)_ = 2.39, *p* = 0.038), *Aif1* (*t*_(10)_ = 4.31, *p* = 0.002) and *MhcII* (*t*_(9)_ = 3.98, *p =* 0.003; Figure 1J). Age significantly reduced Alzheimer's‐associated *App* (*t*_(10)_ = 2.81, *p* = 0.019) and *Sorl1* expression (*t*_(10)_ = 5.14, *p* < 0.001), and increased *Mapt* (*t*_(10)_ = 2.47, *p =* 0.033) and *Abca7* expression (*t*_(9)_ = 2.85, *p =* 0.019; Figure 1K). Finally, age significantly increased expression of the lipid‐related genes *HexB* (*t*_(10)_ = 3.40, *p =* 0.007), *Ch25h* (*t*_(10)_ = 2.72, *p =* 0.022) and *Lpl* (*t*_(10)_ = 2.25, *p =* 0.048; Figure 1L).

Since increased microglial numbers or reduced complexity have previously been shown in rats (Perkins et al. 2018) and in mice (Tremblay et al. 2012) with ageing, we next used confocal imaging to assess if these expected changes were evident in our cohort (Figure 2A,B). In the DG, age increased the numbers of microglia, indicative of a microglial response (*t*_(8)_ = 3.56, *p =* 0.007; Figure 2C) but did not alter the number of branches (Figure 2D), total process length (Figure 2E), or complexity as measured by Sholl (Figure 2F,J). In both the CA1 and CA3 regions of the hippocampus, ageing did not alter the number of microglia (Figure 2C), the number of branches (Figure 2D), total microglial process length (Figure 2E), or complexity (Figure 2H–K). In the ARC, age increased the numbers of microglia (*t*_(8)_ = 3.35, *p* = 0.01; Figure 2A–C) and complexity as measured by mean number of process branches (*t*_(8)_ = 2.63, *p* = 0.03; Figure 2D) but had no effect on total process length (Figure 2E). When assessing ARC microglia complexity using Sholl, there was a significant effect of age on the number of branches for the radius bins (*F*_(3,24)_ = 3.90, *p* = 0.021; Figure 2L,M). Tukey's *post hoc* comparisons demonstrate that microglia from aged ARC were more complex in the 1–10 μm range (*p* = 0.007). These data suggest pronounced morphological changes in ARC microglia in our ageing mice, potentially reflective of early inflammaging.

**FIGURE 2:**
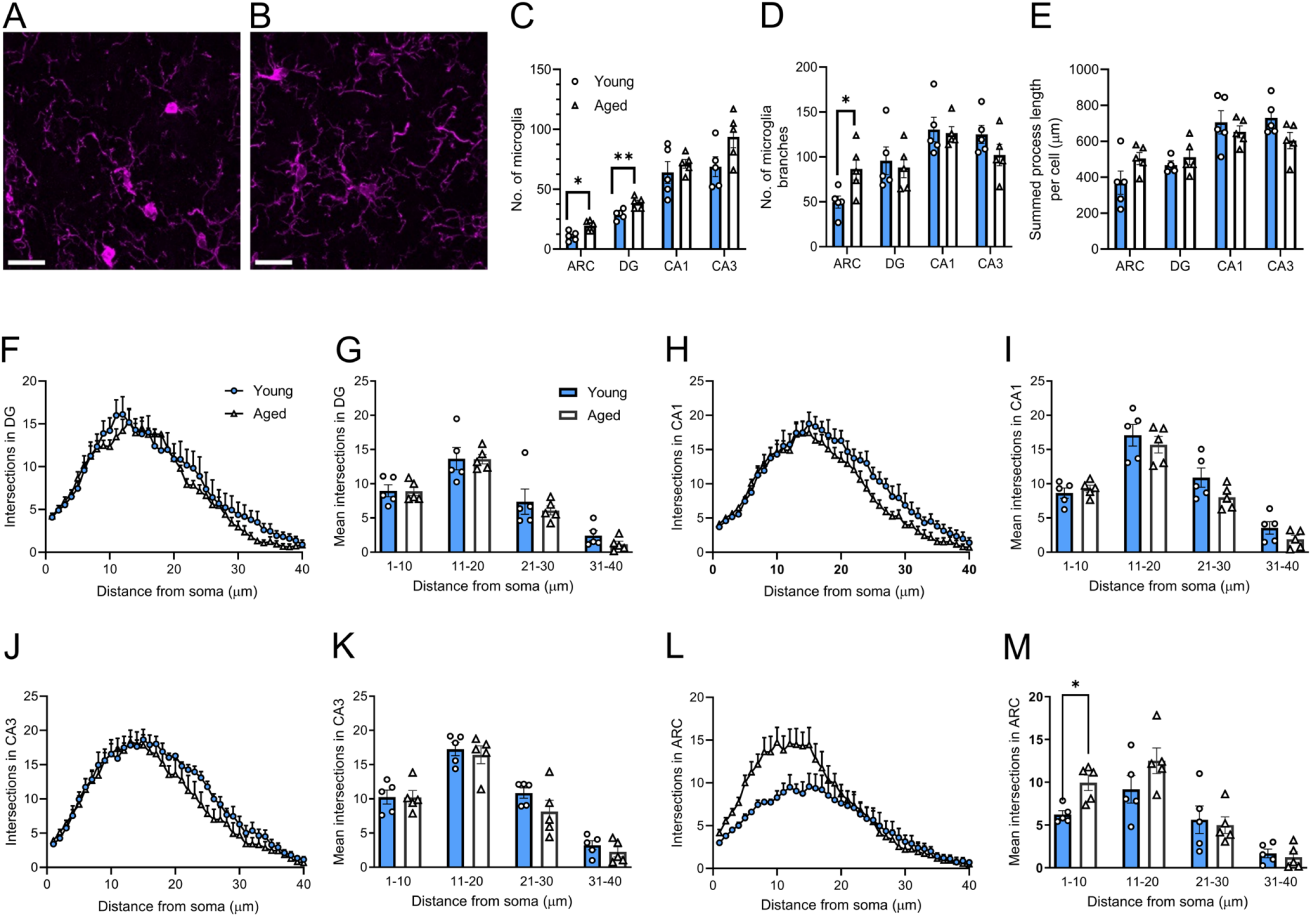
Ageing leads to increased microglial complexity in the arcuate nucleus of the hypothalamus (ARC) and proliferation in the hippocampal dentate gyrus of otherwise healthy mice. Representative images of microglia in the ARC of (A) young and (B) aged mice. (C) Ageing increases the number of microglia in the ARC and dentate gyrus (DG) but not the CA1 or CA3. (D) Ageing increases branch complexity in the ARC but not the DG, CA1, or CA3. (E) Ageing does not alter total microglia process length in any region. Ageing does not alter microglia complexity, as measured by Sholl, in the (F, G) DG, (H, I) CA1, (J, K) CA3 but does increase microglia complexity in the (L, M) ARC. Scale bars = 20 μm. *p < 0.05; **p < 0.01. Data are mean ± SEM. *n* = 5 per group.

### 3.2 Ageing Leads to Accumulation of Autofluorescent Material in the Hippocampus and ARC

In our aged cohort, we observed expected ageing‐related changes, including fewer DCX‐positive neurons in the hippocampus and greater microglial complexity in the ARC. We next assessed whether brain autofluorescence increased with ageing in regions relevant for memory and inflammation as a potential reflection of inflammaging. We therefore assessed the autofluorescent material in the hippocampus and ARC of young and aged animals. In the hippocampus, ageing significantly increased the total volume of autofluorescent material in the DG (*t*_(7)_ = 6.40, *p* < 0.001; Supplementary Figure 2), as well as the CA1 (*t*_(8)_ = 2.39, *p* = 0.044; Supplementary Figure 2) and CA3 regions (*t*_(8)_ = 7.99, *p* < 0.001; Supplementary Figure 2). Ageing also significantly increased the total volume of autofluorescent material in the ARC (*t*_(8)_ = 7.52, *p* < 0.001; Supplementary Figure 2).

### 3.3 Microglial Autofluorescence Increases With Ageing

To assess the potential for this autofluorescence accumulation to correspond to brain changes relevant for inflammaging, we next assessed autofluorescence properties specifically in microglia. In accordance with our previous findings (Slayo et al. 2026), but unlike data obtained from flow cytometry (Burns et al. 2020), all microglia sampled contained at least some autofluorescence material. We found a significant increase in the mean size of the autofluorescent aggregates within microglia in the DG (*t*_(8)_ = 4.88, *p* = 0.001; Figure 3A,B,E). When categorising the autofluorescent aggregates into size bins, there was also a significant effect of age on the size distribution of the material (*F*_(3,24)_ = 14.37, *p* < 0.001; Figure 3F). Young brains had more small autofluorescent particles (*p* = 0.005) while aged brains had more medium (*p* = 0.037), large (*p* = 0.011), and extra‐large autofluorescent aggregates (*p* = 0.002). In the CA1, there was no effect of age on aggregate size (Figure 3G). However, there was a significant effect of age on the size distribution (*F*_(3,24)_ = 5.16, *p* = 0.008; Figure 3H). Young brains had a greater number of small autofluorescent aggregates (*p* = 0.047), while aged brains had a greater number of large particles (*p* = 0.032). In the CA3, there was a significant increase in microglial autofluorescence volume with age (*t*_(8)_ = 5.57, *p* < 0.001; Figure 3I) and a significant effect of age on the size distribution (*F*_(3,24)_ = 13.14, *p* < 0.001; Figure 3J). Again, young brains had a greater number of small autofluorescent granules (*p* = 0.005) while aged brains had a greater number of large (*p* = 0.018), and extra‐large (*p* = 0.001) granules.

**FIGURE 3:**
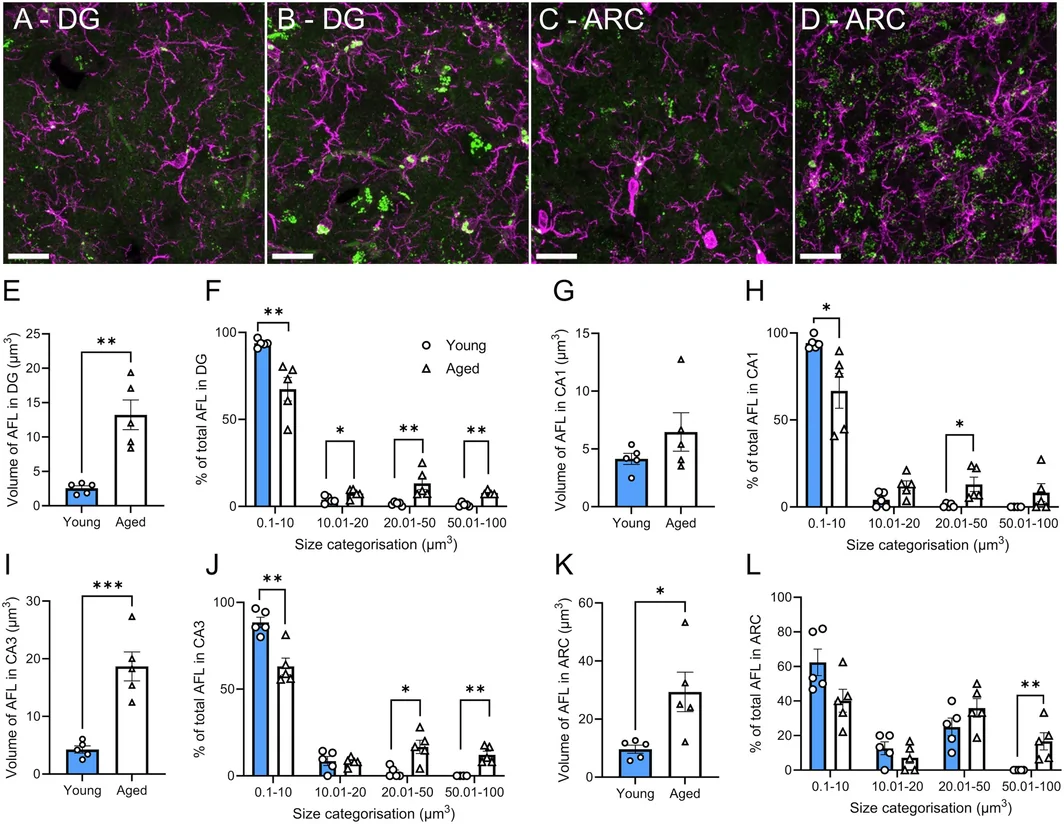
Microglial autofluorescent aggregates increase in size with ageing. (A, B) Representative images of autofluorescent material (detected through 488 nm excitation) and Iba1‐Alexa 647‐stained microglia in the (A) young and (B) aged dentate gyrus region (DG) of the hippocampus, and the (C) young and (D) aged arcuate nucleus (ARC) of the hypothalamus. Microglia from aged mice contain (E) more autofluorescent material and (F) more large autofluorescent aggregates than microglia from young mice in the DG. A similar pattern, as well as fewer of the small autofluorescent aggregates, is seen in the (G, H) CA1, (I, J) CA3, and (K, L) ARC. *p < 0.05. **p < 0.01. ***p < 0.001. Data are mean ± SEM. *n* = 4–5 per group.

In the ARC we also found a significant increase in the mean volume of the autofluorescent material within microglia (*t*_(8)_ = 2.84, *p* = 0.022; Figure 3C,D,K). When binning autofluorescence into small, medium, large, and extra‐large size, there was a significant effect of age on the proportion of autofluorescence within these size bins, similar to that seen in the hippocampus (*F*_(3,24)_ = 4.46, *p* = 0.013; Figure 3L). Tukey's *post hoc* comparisons demonstrated that aged brains had more extra‐large autofluorescent accumulations than young (*p* = 0.010). Together, these findings suggest that microglia accumulate autofluorescence with ageing in the ARC and hippocampus and that aged mice accumulate larger‐sized autofluorescent aggregates.

To determine whether autofluorescence aggregation is associated with microglial activation state, we also assessed the relationship between the mean volume of autofluorescent material within microglia and the microglia branch points (complexity) in the same animal. In the hippocampus (DG, CA1, CA3), we found no association between microglial branching and mean volume of autofluorescence (Supplementary Figure 3). However, in the ARC, microglial branch points showed a significant, moderate relationship to mean autofluorescence volume (*r* = 0.48, *p* = 0.027; Supplementary Figure 3).

### 3.4 Ageing Leads to Altered Autofluorescence Spectral and Lifetime Signatures

To explore potential fluorescence changes within the autofluorescent material with age, we next studied the spectral profile of the autofluorescence using PL spectroscopy. We chose to confine our examination of autofluorescence to the ARC because our findings, above, indicated robust autofluorescent volume differences with age in this region and changes in hippocampus were broadly similar. Figure 4A,B shows examples of small‐ and large‐sized autofluorescent aggregates for spectral analysis, with the white outline indicating the region of interest used. When computing an intensity ratio between two spectral windows, one on either side of the emission peak (Figure 4C), there was a significant main effect of age on the mean intensity ratio (*F*_(2,31)_ = 8.96, *p* < 0.001), with aged, large autofluorescent aggregates having a significantly lower mean intensity ratio (i.e., a relatively red‐shifted emission spectra) than young, small aggregates (*p <* 0.001, Figure 4D).

**FIGURE 4:**
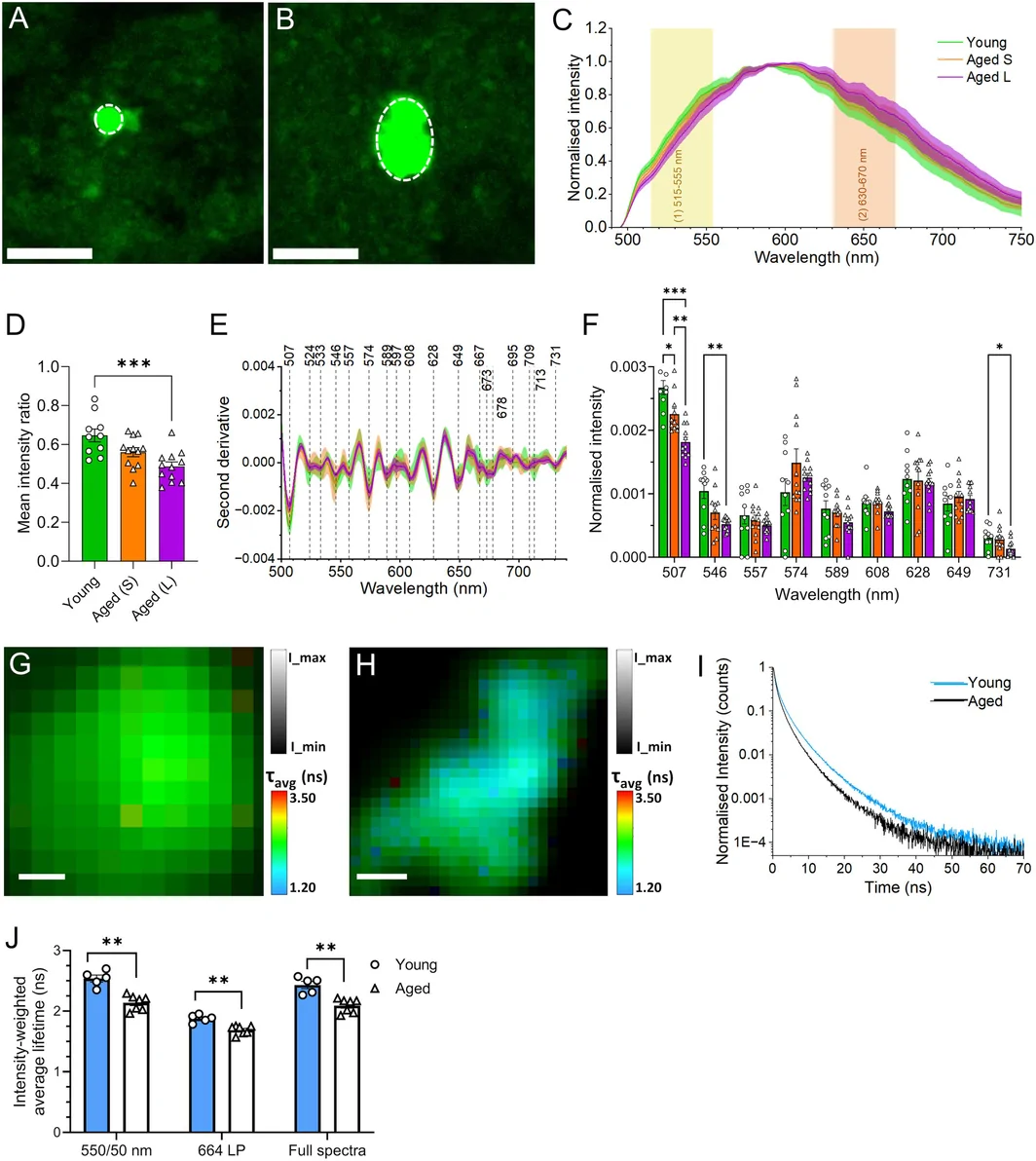
Ageing leads to altered spectral profiles and fluorescence lifetime of brain autofluorescence. (A, B) Representative images of (A) a small autofluorescent aggregate analysed for spectra in young tissue and (B) a large aggregate analysed in aged tissue. (C) Normalised intensity of each group at a range of spectral emission wavelengths. (D) Mean ratio of intensities between 515 and 555 nm and 630–670 nm as shown in (C). (E) Second derivatives of the autofluorescence spectra of each group identifying hidden peaks in the emission spectrum. (F) Comparison of the magnitude of the second derivative spectra for the peaks appearing in > 90% of animals. (G, H) Representative fluorescence lifetime images of (G) young and (H) aged, large autofluorescence in the arcuate nucleus of the hypothalamus at the full spectrum collected in the 500–750 nm wavelength range. Monochromatic scale = events (counts), polychromatic scale = intensity‐weighted average lifetime. (I) Representative fluorescence decay curves of autofluorescent granules in young and aged brains. (J) Intensity‐weighted average lifetime at the 525–575 nm, 664 nm long‐pass and full spectrum emission window is shorter for autofluorescence in aged brains than in young. S = small, L = large. (A, B) scale bar = 5 μm, (G) scale bar = 0.2 μm, (H) scale bar = 0.5 μm. (A–F) Data are mean ± SEM. *n* = 10–12 per group. (G–J) Data are mean ± SEM. *n* = 4–5 per group. *p < 0.05. **p < 0.01. ***p < 0.001.

Given that ageing‐associated autofluorescence aggregates likely represent chemically complex and evolving structures, resolving their spectral substructure is critical for defining a functional biophotonic phenotype of ageing. The broad autofluorescence spectra we observed are consistent with the coexistence of multiple endogenous fluorophores within the autofluorescence aggregates. To identify individual peaks that contributed to the overall emission spectra, we calculated the second derivative of the smoothed and normalised spectra (Figure 4E) and identified 18 individual peaks ranging from 507 to 731 nm (see Supplementary Figure 4). Of these, we selected only the peaks that were present in > 90% of the animals to compare the magnitude of the second derivative. This resulted in nine autofluorescent peaks, occurring at 507, 546, 557, 574, 589, 608, 628, 649, and 731 nm. There was a significant interaction between age and size for normalised emission intensity (*F*_(16,243)_ = 3.03, *p* < 0.001; Figure 4F). The magnitude of the second derivative for the 507 nm peak was higher for young, small aggregates than for aged, small aggregates (*p* = 0.026) and aged, large aggregates (*p* < 0.001). Aged, small aggregates displayed a less prominent second derivative peak at 507 nm than aged, large aggregates (*p* = 0.004). Meanwhile, the 546 nm peak was less prominent in the autofluorescence from aged, large compared to young (*p* = 0.004). The 731 nm peak displayed a lower magnitude in the second derivative spectra of aged, large aggregates compared to young autofluorescent aggregates (*p* = 0.047).

Fluorescence lifetime provides information about biological state that intensity imaging cannot, as it is sensitive to pH levels, ion concentration, protein binding state, and other cellular environmental factors (Blacker et al. 2023; Linders et al. 2022). Therefore, we sought to determine whether our biophotonic fingerprint could also capture functional, environment‐sensitive changes in brain autofluorescence with age. We employed FLIM to measure the radiative lifetime of the autofluorescent material in the ARC of young and aged brains (Figure 4G,H). We measured the intensity‐weighted fluorescence lifetime across the entire emission spectrum (500–750 nm) and in spectral regions corresponding to the windows used to determine spectral ratios. Autofluorescence in the aged mice had a significantly shorter lifetime at all emission windows including the 525–575 nm (*t*_(10)_ = 5.40, *p* < 0.001), 664 nm long‐pass (*t*_(10)_ = 4.67, *p* < 0.001), and full spectrum (*t*_(10)_ = 4.75, *p* < 0.001; Figure 4I,J). For example, in the full spectrum, autofluorescence from young mice averaged 2.43 ± 0.13 ns, while autofluorescence from aged mice averaged 2.09 ± 0.11 ns. This illustrates a reduction in the autofluorescence lifetime that occurs with ageing, which can be paired alongside the spectral red‐shift for a more robust imaging profile of the ageing brain, indicating ageing‐related changes in brain autofluorescence.

### 3.5 Ageing Alters the Lipid Profile in Plasma, and This Profile Significantly Correlates With Brain Autofluorescence

Since we had found large autofluorescence aggregates in ageing mouse brains to have a unique spectral signature, we next attempted to identify peripheral changes that reflected this signature. Therefore, we conducted LC–MS to generate a lipidomic analysis on plasma samples from the same mice as for our autofluorescence analysis.

Since our spectral data had identified a clear group difference where large, red‐shifted autofluorescence aggregates were present in all aged mice and no young mice, we first identified those lipids that were significantly different between the aged and young groups. Of all lipids, 16 were upregulated and six were downregulated in ageing with a significant change (FC > 1.5, FDR < 0.1; Figure 5 and Table 1).

**FIGURE 5:**
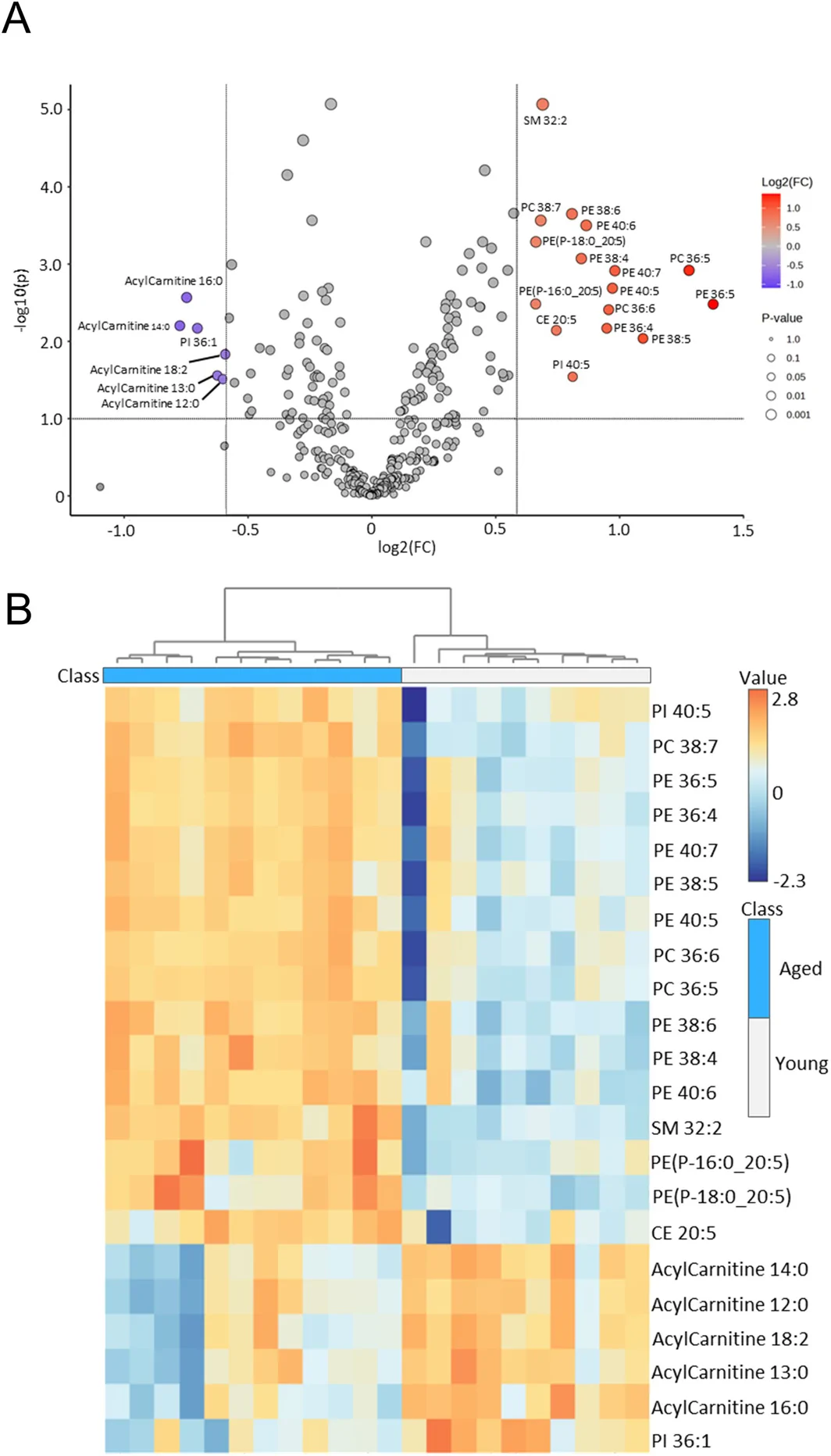
Ageing‐related plasma lipid changes. (A) Volcano plot of plasma lipidomics analysis showing differentially expressed lipids between young and aged mice. Lipids to the left of x = −0.585 and above y = 1.0 (blue) are significantly decreased with age, while lipids to the right of x = 0.585 and above y = 1.0 (red) are significantly and strongly enriched with age. (B) Heat map of the 22 significantly and strongly different plasma lipid species between young and aged mice identified by the volcano plot. Each column represents a single animal. Each coloured cell corresponds to a concentration value as determined by mass spectrometry. Orange cells represent a lipid higher in abundance in ageing relative to the young samples, blue cells represent a lipid that is lower in abundance in ageing compared to the average for that sample. Dendrogram indicates clustering of samples with similar lipid concentration.

**TABLE 1:** Lipids identified by volcano plot analysis as significantly and moderately changed (fold change > 1.5, FDR < 0.1) between young and aged plasma.

	FC	log2(FC)	raw.pval	−log10(p)
SM 32:2	1.62	0.69	0.00	5.07
PE 38:6	1.75	0.81	0.00	3.65
PC 38:7	1.60	0.68	0.00	3.57
PE 40:6	1.82	0.87	0.00	3.50
PE(P‐18:0_20:5)	1.58	0.66	0.00	3.29
PE 38:4	1.80	0.85	0.00	3.07
PC 36:5	2.43	1.28	0.00	2.92
PE 40:7	1.97	0.98	0.00	2.92
PE 40:5	1.96	0.97	0.00	2.69
AcylCarnitine 16:0	0.60	−0.75	0.00	2.57
PE(P‐16:0_20:5)	1.58	0.66	0.00	2.48
PE 36:5	2.60	1.38	0.00	2.48
PC 36:6	1.94	0.96	0.00	2.41
AcylCarnitine 14:0	0.58	−0.78	0.01	2.20
PE 36:4	1.93	0.95	0.01	2.17
PI 36:1	0.61	−0.70	0.01	2.17
ce 20:5	1.68	0.75	0.01	2.14
PE 38:5	2.14	1.09	0.01	2.04
AcylCarnitine 18:2	0.67	−0.59	0.02	1.83
AcylCarnitine 13:0	0.65	−0.62	0.03	1.56
PI 40:5	1.75	0.81	0.03	1.54
AcylCarnitine 12:0	0.66	−0.60	0.03	1.51

*Note:* Lipids that are less abundant with age are indicated by grey, while those that are more abundant with age are white.

Using a simple linear regression on each lipid, 12 lipids were significantly associated with brain autofluorescence spectra independently of age (*p* < 0.05, Table 2), while 10 were not (Supplementary Table 2). Two of these lipids survived the Bonferroni correction for multiple comparisons placing statistical significance at *p* < 0.0023. These were SM 32:2 (*F*_(1,20)_ = 13.0, *R* = 0.624, *R* ^2^ = 0.390, *p* = 0.0018) and PE 38:6 (*F*_(1,20)_ = 12.78, *R* = 0.628, *R* ^2^ = 0.394, *p =* 0.0019; Table 2). This demonstrates that as the autofluorescence emission spectral profile becomes more red‐shifted, there is increased SM 32:2 and PE 38:6 in the plasma.

**TABLE 2:** Simple linear regression between brain spectral ratio and those plasma lipids significantly and moderately altered (fold change > 1.5, FDR < 0.1) with ageing (Bonferroni‐corrected significance of *p* < 0.002); light grey.

Lipid	Unstd. coefficients		*t*	Sig.
	Beta	Std. error		
SM 32:2	−0.63	0.18	−3.61	0.0018
PE 38:6	−0.47	0.13	−3.58	0.0019
PE 38:4	−0.38	0.12	−3.08	0.006
PC 38:7	−0.47	0.17	−2.78	0.011
PE 40:6	−0.36	0.13	−2.82	0.011
PE(P‐18:0_20:5)	−0.51	0.18	−2.77	0.012
PE 40:7	−0.26	0.10	−2.47	0.023
AcylCarnitine 16:0	0.33	0.14	2.30	0.032
PE 40:5	−0.24	0.10	−2.29	0.033
PE 36:4	−0.20	0.091	−2.19	0.041
AcylCarnitine 18:2	0.32	0.15	2.18	0.042
PC 36:5	−0.17	0.080	−2.11	0.048

*Note:* Those found to be statistically significantly changed without correcting for multiple comparisons are indicated in white.

### 3.6 Independent Validation That Ageing Alters the Autofluorescence Spectral Profile and the Plasma Lipid Profile, and That These Lipids Significantly Correlate With Brain Autofluorescence

Since we had identified two lipids potentially associated with brain autofluorescence changes with ageing (SM 32:2 and PE 38:6), we next tested if these lipids were reproducibly associated with brain autofluorescence changes in a different cohort. We therefore examined spectral ratios and lipidomic analyses in a separate cohort of ageing mice culled and processed approximately 6 months after the first. We chose to examine the same second derivative peaks as the original cohort to determine their replicability. These data revealed that the same spectral red‐shift is observed in the aged brain autofluorescence of the validation cohort (Figure 6A,B). When computing the intensity ratio between blue and red spectral windows, there was, again, a significant main effect of age on the mean intensity ratio (*F*_(2,15)_ = 1.25, *p* = 0.025), with aged, large autofluorescent aggregates having a significantly lower mean intensity ratio (i.e., red‐shifted) than young aggregates (*p* = 0.019, Figure 6B) as with the first cohort. When selecting the hidden peaks present in more than 90% of the animals, most of the nine from the first cohort were still present in the validation cohort with the new addition of 524 nm, 667 nm, and 678 nm and omission of 557 nm. That is, the first three were now present in more than 90% of the animals and the last (557 nm was not) (Figure 6C,D). There was a significant interaction between size (age) and wavelength for normalised intensity across all peaks identified in the second derivative spectrum (*F*_(20,148)_ = 3.05, *p* < 0.001; Figure 6D). As in the first cohort, the 507 nm peak was less intense in aged, large autofluorescence than in young (*p* = 0.006) but not significantly different to the aged, small. The 524 nm peak, not amongst the nine peaks in the original cohort, was significantly less intense in the aged, large aggregates than in the young, small (*p* = 0.039). At 608 nm, young had significantly lower intensity relative to aged, small (*p* = 0.028) and aged, large (*p* = 0.013). We also observed a significantly greater intensity in the aged, large aggregates at the 678 nm (*p* = 0.02) and 731 nm peak (*p* < 0.001) relative to young and aged, small, respectively. However, these were not statistically significant in the first cohort. When including the tumour‐bearing animal, a similar spectral red‐shift and peak pattern is observed (see Supplementary Figure 5). Together, these support the overall observed spectral red‐shift in aged, large aggregates (Figure 6B).

**FIGURE 6:**
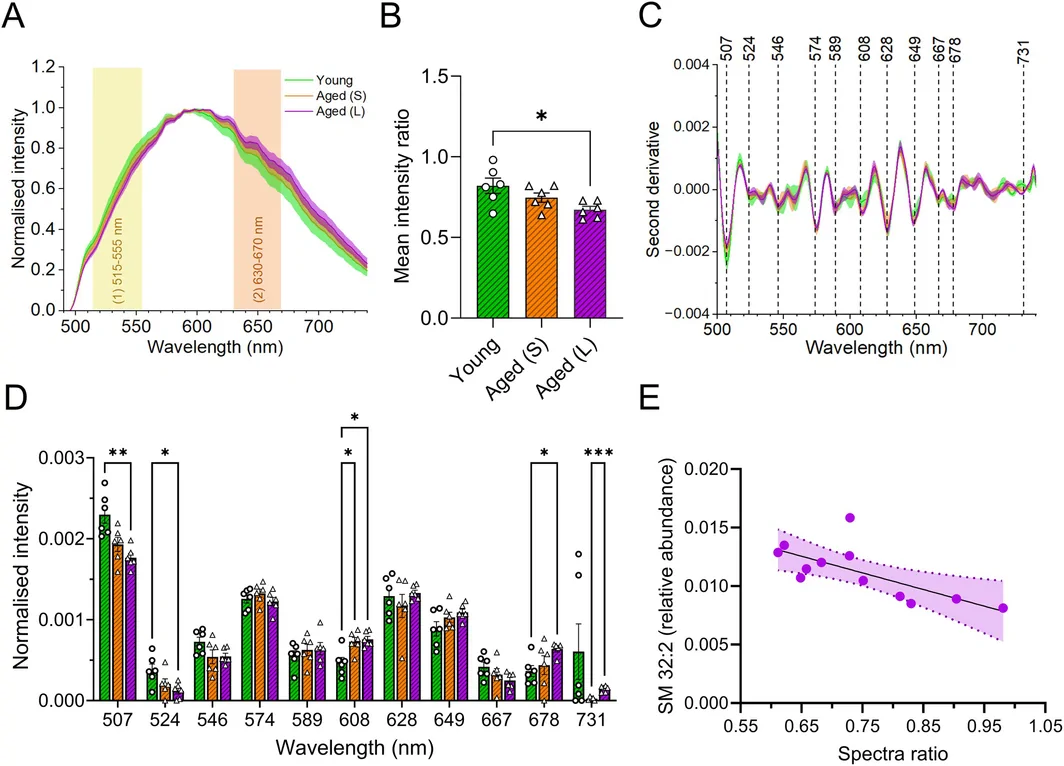
The brain autofluorescence spectral changes and relationship to plasma sphingomyelin (SM) 32:2 is replicable in a second cohort. (A) Normalised intensity of each group of the validation cohort at a range of spectral emission wavelengths. (B) Mean ratio of intensities between the emission at 515–555 nm and 630–670 nm as shown in (A). (C) The second derivatives of each group identifying hidden peaks in the emission spectrum. (D) Comparison of intensities at each identified peak. (E) Plasma sphingomyelin 32:2 (SM 32:2) predicts changes in brain autofluorescence spectral shift in the validation cohort, accounting for 57% of the variance (*R* = 0.755, *R* ^2^ = 0.570). For ease of interpretation, sum‐normalised, log_10_‐transformed plasma SM 32:2 values are back‐transformed to linear scale to represent relative abundance only for visualisation purposes. Higher abundance values indicate greater levels of lipid abundance, and lower values indicate reduced levels of lipid abundance. Higher spectra ratios indicate a blue‐shifted emission peak, and lower ratio values indicate a red‐shifted emission peak. *p < 0.05. **p < 0.01. ***p < 0.001. Data are mean ± SEM. *n* = 6–7 per group.

Processed lipid intensities were used to run a simple linear regression between their spectral profile and the two lipid candidates identified from our first analysis, SM 32:2 and PE 38:6. There was no significant relationship between peripheral PE 38:6 and brain autofluorescence spectra (*F*_(1,10)_ = 0.51, *p* = 0.49, Supplementary Table 3). However, SM 32:2 had a significant relationship with the brain autofluorescence spectral profile as in the earlier experiment, accounting for 57% of the variance in brain autofluorescence spectra (*F*_(1,10)_ = 13.26, *R* = 0.755, *R* ^2^ = 0.570, *p* = 0.005; Figure 6E and Supplementary Table 3). In the case with the tumour‐bearing animal included in the analysis, the same pattern is present with SM 32:2 accounting for 57% of the variance (*F*_(1,11)_ = 14.56, *R* = 0.755, *R* ^2^ = 0.570, *p* = 0.003, Supplementary Table 3). Again, this demonstrates that more red‐shifted brain autofluorescence emission spectra are associated with increased SM 32:2 in the plasma, underlining SM 32:2 as a potential peripheral marker of ageing brain autofluorescence spectra across animal cohorts.

As an exploratory measure, we also assessed whether the nine lipids identified in the first cohort as statistically significantly different at *p* < 0.05 but not surviving the Bonferroni correction were associated with brain spectra ratios in the validation cohort without Bonferroni correction. (see Supplementary Table 4). Two of these lipids were not detected in the validation cohort (PE 40:6 and AcylCarnitine 18:2). However, three lipids showed a repeated significant relationship to brain spectra: AcylCarnitine 16:0 (*F*_(1,10)_ = 7.30, *p* = 0.022), PE 36:4 (*F*_(1,10)_ = 6.93, *p* = 0.025), and PE 38:4 (*F*_(1,10)_ = 4.97, *p* = 0.050). As the brain autofluorescence emission spectral peak was more red‐shifted, there was less plasma AcylCarnitine 16:0 and more plasma PE 38:4 and PE 36:4.

## 4 Discussion

In this study, we aimed to define ageing‐related changes in microglial autofluorescence in the hippocampus and ARC, and whether these were associated with spectral and lifetime signatures that can be identified with plasma lipid profiles. First, we established that our mice had a typical ageing brain phenotype. As shown in previous literature, this is marked by reduced numbers of immature neurons, greater microglia numbers, and greater tissue autofluorescence in the brain (Culig et al. 2022; Wong 2013), all of which we recapitulate here. Similar to previous studies in rodents and humans, we also see ageing‐related changes to central expression of genes indicative of disease‐associated states, or at least inflammaging, including genes relating to inflammation (*Itgam, Tlr4, Mhc‐II, Aif1*), Alzheimer's disease (*Sorl1*, *Mapt*, *Abca7*), and lipid pathways (*Hexb, Ch25h, Lpl*) (Boche and Gordon 2022; Kim 2018; Long et al. 2022).

In association with this typical and well‐established ageing‐brain profile, we revealed that ageing brains have a propensity to accumulate larger‐sized aggregates in all hippocampal regions assessed (DG, CA1, and CA3) and the ARC. When assessing the emission spectra of autofluorescence aggregates of varying sizes in young and aged brains, we identified consistent eight spectral peaks common to both age groups. Notably, the red‐shifted intensity ratio we observed consistently distinguished aged from young brains, suggesting the potential utility of an overall emission spectral shift as a future spectral biomarker of ageing. To our knowledge, this is the first study to systematically compare spectral autofluorescence signatures between young and aged brains. We also found that aged tissue exhibited consistently shorter intensity‐weighted average lifetimes across all three detection windows compared with young tissue, indicating altered autofluorescence decay dynamics with age. Supporting this interpretation, a previous study examining autofluorescent inclusions in female *APPswe:PS1dE9* transgenic mice reported that both plaque‐surrounding and plaque‐free cells displayed shorter autofluorescence lifetimes than their wild‐type counterparts (Gómez et al. 2018). This suggests that changes in autofluorescence lifetime reflect systemic neurodegenerative physiological alterations and are not solely attributable to the presence of disease‐specific markers such as amyloid‐beta plaques.

One working hypothesis is that these differences likely reflect fundamental physiological changes that accompany ageing. With advancing age, increasing iron accumulation promotes reactive oxygen species formation, which in turn drives lipid peroxidation and the buildup of cross‐linked, undegradable biomaterials within lysosomal and autophagosomal compartments of post‐mitotic cells (as reviewed in (Yin 1996)). Defective lysosomal acidification–a hallmark of both ageing and neurodegenerative diseases such as Alzheimer's (Lo and Zeng 2023)–further impairs autophagic clearance, exacerbating the accumulation of autofluorescent material (Escrevente et al. 2021). Collectively, these processes may illustrate how ageing‐related alterations in cellular maintenance mechanisms, particularly lysosomal and autophagic dysfunction, can create a self‐perpetuating cycle of oxidative stress and impaired degradation that underlies the pathogenesis of ageing‐related diseases. Barbosa and colleagues succinctly described this mechanism of ageing‐related disease as an imbalance between increasing oxidative stress and declining autophagic capacity over the lifespan (Barbosa et al. 2018).

This observation is particularly significant, as ageing‐related increases in autofluorescence have been attributed to alterations in autophagic and lysosomal processing (Burns et al. 2020). In our study, we observed a pronounced age‐dependent increase in microglial autofluorescence across the DG, CA1, CA3, and ARC. Burns and colleagues demonstrated that microglial autofluorescence is not influenced by inhibition of Fc‐receptor or TREM2‐mediated phagocytosis but rises markedly when autophagy and lysosomal degradation are disrupted (Burns et al. 2020). Consistent with this, our data show that young microglia predominantly contain small autofluorescent aggregates, whereas aged microglia accumulate more medium, large and extra‐large ones. Together with existing literature and the observed age‐related downregulation of the endolysosomal trafficking gene *Sorl1*, these findings raise the possibility that aged microglia may progressively accumulate autofluorescent material because of multiple, lifespan‐associated processes. Namely, this likely reflects both cumulative oxidative stress–leading to the buildup of oxidised lipids and proteins (Yin 1996)–and a concurrent decline in autophagic efficiency (Tsong et al. 2023), resulting in impaired clearance and build‐up of autofluorescent waste products (Burns et al. 2020). In this context, the rate of autofluorescent material accumulation may eventually exceed the cell's capacity to degrade it, establishing a self‐reinforcing cycle in which undegraded material further disrupts lysosomal function and autophagy.

It is possible that one of the observed physiological changes–disruptions in lysosomal acidification, known to accompany both ageing and neurodegenerative disorders such as Alzheimer's disease (Escrevente et al. 2021)–contributes to the observed spectral and lifetime alterations. Although fluorescence lifetime has not previously been measured across varying lysosomal pH states, studies in HeLa cells demonstrate that increasing extracellular pH leads to progressively longer fluorescence lifetimes (Schmitz et al. 2021). Similarly, pH variation influences emission spectra: in *Prevotella intermedia* , higher pH levels enhance blue emission while reducing red emission (Hope et al. 2016). In the context of our findings, these observations suggest that age‐related shifts in lysosomal acidity could partially underlie the overall red‐shifted spectra and shorter lifetimes we detected in aged microglia. More broadly, the ageing brain represents a highly complex biochemical environment in which multiple processes–including lysosomal acidification, impaired autophagy, and lipid peroxidation–likely converge to alter the photophysical behaviour of endogenous fluorophores. This complex interaction may account for why we observed a reproducible overall spectral shift in our validation cohort but minor differences in individual emission peaks. Crucially, these hypotheses remain untested. Further investigation into how these biochemical dynamics shape autofluorescence spectral and lifetime properties is needed and may ultimately enable the identification of distinct photophysical biomarker signatures for different neuropathological states in the future.

Notably, here we identified 22 plasma lipids that significantly change with age and, for the first time, we demonstrate that at least two of these (SM 32:2 and PE 38:6) also reflect ageing‐related changes in brain autofluorescence spectra. When testing the reliability of the two successful markers, SM 32:2 was significantly associated with brain autofluorescence spectra in a separate validation cohort. SM 32:2 is a sphingomyelin that increases across the female lifespan independent of lipid‐lowering medication use (Slade et al. 2021). Broadly, SMs have been implicated in inflammatory and immune responses, vascular homeostasis, cancer, and diabetes (Zeidan and Hannun 2007). Levels of SMs are associated with memory impairment in ageing (Mielke et al. 2010), and their increase causes lysosomal damage and autophagy dysfunction in lysosomal storage disorders (Gabandé‐Rodríguez et al. 2014). Our lipid species of interest, SM 32:2, is positively associated with ageing in the literature (Weir et al. 2013) as well as subclinical atherosclerosis, a disease characterised by chronic vascular inflammation (Sojo et al. 2023), suggesting that this lipid might reflect systemic lipidomic adaptations to pathological and ageing‐related inflammatory stress. However, plasma levels of SM 32:2 are significantly higher in female than male humans (Weir et al. 2013), possibly limiting its future biomarker potential to only females. Female mice also show higher neuroinflammation compared to males even at young ages (Cyr and de Rivero Vaccari 2023; Hu et al. 2013). As our work was conducted here only in female mice, it will be important to repeat this study in a male cohort to understand how sexual dimorphism influences the relationship between brain autofluorescence and peripheral changes. Similarly, generalisability will need to be tested in other models and with greater sample sizes. Thus, while rising plasma SM levels may reflect normal ageing‐associated inflammation, such increases are likely to be further exacerbated in ageing‐related pathological conditions and SM 32:2 may be uniquely related to this state in females.

## 5 Conclusion

This study establishes, for the first time, a reproducible photophysical fingerprint of brain ageing by integrating autofluorescence spectroscopy and lifetime imaging with plasma lipidomics. Our findings reveal that the ageing brain exhibits a distinct shift in autofluorescence spectral and lifetime decay properties, likely reflecting lysosomal and autophagic decline, oxidative stress, and lipid remodelling–core hallmarks of inflammaging. Crucially, these autofluorescence signatures correlate with specific circulating lipid species, particularly SM 32:2, suggesting a co‐occurring bidirectional relationship between central and peripheral biochemical ageing processes.

Our data introduce the concept of a “biophotonic phenotype” of ageing–a measurable optical signature that likely reflects cellular metabolic state, lysosomal health, and lipid homeostasis. This offers a fundamentally new dimension to ageing biology, bridging molecular pathology with optical physics. Already, in vivo hyperspectral retinal imaging of autofluorescence in humans has successfully differentiated individuals with Alzheimer's disease from healthy controls (Hadoux et al. 2019). Translationally, these insights open the path toward future non‐invasive optical biomarkers of early neurodegenerative vulnerability. Integrating such optical data with AI‐driven spectral analysis and lipidomic profiling could enable individualised, physiology‐based monitoring of brain ageing and preclinical neurodegeneration–well before structural or cognitive decline becomes detectable. In sum, our work defines a new class of potential future ageing biomarkers grounded in the photophysical behaviour of endogenous fluorophores and their systemic biochemical correlates. This work establishes the foundation for future studies to track, quantify, and ultimately intervene in the earliest physiological shifts linking normal ageing to neurodegenerative disease.

## Author Contributions

M.S., K.M.Q., B.D.R., S.J.S. designed the study; M.S., L.V., S.X., P.S. performed the experiments and analysed the data; M.S. and S.J.S. wrote the manuscript.

## Funding

This project was funded by an Australian Research Council (ARC) Discovery Project (DP230101331) to S.J.S. and B.R. as well as funding from a European Union (EU) Joint Program on Neurodegenerative Disease (JPND) Grant: (SOLID JPND2021‐650‐233) to S.J.S. S.J.S. is also supported by a National Health and Medical Research Council (NHMRC) Ideas Grant (2019196). B.R. is also supported by an ARC DECRA (DE200100985), an Australian Academy of Sciences (JG Russell Award) and an RMIT University Vice Chancellor's (VC) Fellowship. K.M.Q. was supported by an RMIT VC Fellowship. M.S. is supported by an RMIT University VC's Ph.D. Scholarship.

## Ethics Statement

We conducted all experiments in accordance with the Australian Code of Practice for the Care and Use of Animals for Scientific Purposes, with approval from the RMIT University Animal Ethics Committee. Application Numbers: AEC22406, AEC24986 and AEC23208.

## Consent

The authors have nothing to report.

## Conflicts of Interest

The authors declare no conflicts of interest.

## Supporting information


**Supplementary Figure 1:** A schematic representation of time‐correlated single photon counting (TCSPC)‐based scanning fluorescence lifetime imaging. A pulsed 485 nm laser is focused on a region‐of‐interest around the autofluorescent aggregate, which is scanned and dedicated using timing electronics and a single point detector. Different emission windows are acquired by manually changing a cube containing a mirror and filters. DBS, dichroic beamsplitter; TCSPC, time‐correlated single photon counting. Figure created with BioRender.com; Toronto, Canada.
**Supplementary Figure 2:** Ageing leads to accumulation of autofluorescent material in the hippocampus and ARC. (A–D) compared to young, aged animals have more autofluorescent material detected through 488 nm excitation in the (A) dentate gyrus (DG), (B) CA1 region of the hippocampus, (C) CA3 region, and (D) arcuate nucleus of the hypothalamus (ARC). (E–L) Representative images of autofluorescent material in the (E) young and (F) aged DG, (G) young and (H) aged CA1, (I) young and (J) aged CA3, and (K) young and (L) aged ARC. (M, N) autofluorescent material colocalises within Iba1‐positive microglia labelled with Alexa 647 in (M) young and (N) aged ARC. Scale bars = 20 μm. *p < 0.05, ***p < 0.001. Data are mean ± SEM. *n* = 4–5.
**Supplementary Figure 3:** Microglia autofluorescence volume is associated with branching complexity in the ARC but not the hippocampus. The number of microglia branches are not associated with the volume of microglial autofluorescence in the (A) DG, (B) CA1, or (C) CA3. (D) In the ARC, there is a significant, moderate relationship between microglial branching and volume of microglial autofluorescence (*p* = 0.027). Data points represent the mean values of individual animals (*n* = 10). Blue circles = young, white triangles = aged.
**Supplementary Figure 4:** Second derivative analysis of the spectral emission showing all identified peaks including those with < 90% presence in all animals in (A) original cohort and (B) validation cohort. (C) A comparison of intensities at all identified peaks in the original cohort. Like the selective analysis for the original cohort, there was still a significant interaction between age and size for normalised intensity across all peaks identified in the second derivative spectrum (*F*_(34,555)_ = 1.89, *p* = 0.002). At 507 nm, young animals still had significantly higher intensities than aged, small (*p* = 0.026) and aged, large (*p* < 0.001) autofluorescence. Similarly at this peak, aged, small autofluorescent aggregates were still more intense than aged, large (*p* = 0.004). Young aggregates also had a significant higher intensity than aged, large autofluorescent aggregates at 546 nm (*p* = 0.004) and 731 nm (*p* 0.047). (D) A comparison of intensities at all identified peaks in the validation cohort. Like the selective analysis for the validation cohort, there was still a significant interaction between age and size for normalised intensity across all peaks identified in the second derivative spectrum (*F*_(34,269)_ = 2.12, *p* < 0.001). Young aggregates had a significantly higher intensity than aged, large aggregates at 507 nm (*p* = 0.006) and 524 nm (*p* = 0.039). At 608 nm, young aggregates had a significantly lower intensity than aged, small (*p* = 0.028) and aged, large (*p* = 0.013) aggregates but there was no significant difference at 731 nm in this second cohort. *p < 0.05. **p < 0.01. *** *p* < 0.001. Data are mean ± SEM. *n* = 10–12 per group and 6 per group in each cohort, respectively.
**Supplementary Figure 5:** A similar spectral pattern is observed with age when including the tumour‐bearing animal (indicated by the red data point). (A) Mean ratio of intensities between the emission at 515–555 nm and 630–670 nm. (B) Comparison of intensities at each identified peak. Young aggregates had a significantly higher intensity than aged, large at 507 nm (*p* = 0.012) and 524 nm (*p* = 0.043). Aged, large aggregates had a significantly higher intensity than young (*p* = 0.009) and aged, small (*p* = 0.012) aggregates at 608 nm and 731 nm, respectively. *p < 0.05. **p < 0.01. Data are mean ± SEM. *n* = 6–7 per group.

**Supplementary Methods 1:** Code used to analyse autofluorescence within microglia from confocal Z‐stacks.

**Supplementary Table 1:** Taqman primer details.
**Supplementary Table 2:** Age‐associated plasma lipids without significant associations with brain spectral ratios (fold change > 1.5, FDR < 0.1).
**Supplementary Table 3:** Simple linear regression with brain spectra ratio and the two candidate plasma lipid intensities in the validation cohort with the tumour‐bearing animal excluded (grey) and included (white).
**Supplementary Table 4:** Simple linear regression of the remaining lipids from the 9 original cohort lipids (non‐significant after Bonferroni correction) assessed in the validation cohort (*p* < 0.05). PE 40:6 and AcylCarnitine 18:2 were not detected in this validation cohort.

## Data Availability

The data that support the findings of this study are available in the Supplementary Materials of this article or from the corresponding author upon reasonable request.
