# Supplementary material for: Establishing a Biophotonic Signature of Brain Ageing
Source: Aging Cell. 2026 Nov 1;25(11):e70790. doi: 10.1111/acel.70790 (PMC13724186; doi:10.1111/acel.70790)
Supplement: Supplementary file 1 [file ACEL-25-e70790-s001.docx]

**Supplementary Methods**

Supplementary Methods 1. Code used to analyse autofluorescence within microglia from confocal Z-stacks.

*// 1. Pre-analysis*
 *// 1.1. Initialise GPU*
 run("CLIJ2 Macro Extensions", "cl_device=");
 Ext.CLIJ2_clear();

 *// 1.2 Define Output directory path*
 OutputDir = "D:/AFL analysis/";

 *// 1.3 Get image title and metatdata*
 imageTitle = getTitle();
 metaData = getMetadata("Info");
 getVoxelSize(pixelWidth, pixelHeight, pixelDepth, unit);
 frameInterval = Stack.getFrameInterval();

*//2. Processing autofluorescence (AFL) channel (channel #1 in 2-channel stacks)*

 *// 2.1 Smoothing and background subtraction in GPU*
 *// Send channel to GPU*
 selectWindow(imageTitle);
 Stack.setChannel(1);
 Ext.CLIJ2_pushCurrentZStack(imageTitle1);

 *// Smooth in GPU*
 Ext.CLIJ2_median3DSphere(imageTitle1, AFL_Smoothed1, 1.0, 1.0, 0.3);
 Ext.CLIJ2_gaussianBlur3D(AFL_Smoothed1, AFL_Smoothed2, 0.8, 0.8, 0.3);
 *// Subtract Gaussian Background in GPU*
 Ext.CLIJx_subtractBackground3D(AFL_Smoothed2, BGsub_AFL, 40.0, 40.0, 16.6);

 *// 2.2. Send smoothed and background subtracted AFL stacks back to ImageJ and apply metadata*
 Ext.CLIJ2_pull(AFL_Smoothed1);
 rename("Smoothed AFL");
 setMetadata("Info", metaData);
 run("Properties...", "unit="+unit+" pixel_width="+pixelWidth+" pixel_height="+pixelHeight+" voxel_depth="+pixelDepth);
 Stack.setFrameInterval(frameInterval);

 Ext.CLIJ2_pull(BGsub_AFL);
 rename("BG-subtracted AFL");
 setMetadata("Info", metaData);
 run("Properties...", "unit="+unit+" pixel_width="+pixelWidth+" pixel_height="+pixelHeight+" voxel_depth="+pixelDepth);
 Stack.setFrameInterval(frameInterval);

 *// 2.3 AFL thresholding and label generation*
 *// Generate AFL binary mask using MaxEntropy autothresholding*
 run("Duplicate...", "duplicate");
 setOption("ScaleConversions", **true**);
 setAutoThreshold("MaxEntropy dark 16-bit stack no-reset");
 setOption("BlackBackground", **true**);
 run("Convert to Mask", "method=MaxEntropy background=Dark calculate black create");
 rename("AFL TH mask");
 run("Duplicate...", "duplicate");
 run("Divide...", "value=255 stack");
 run("16-bit");
 rename("Binary AFL mask");

 *// Apply mask to smoothed AFL stack*
 imageCalculator("Multiply create stack", "Smoothed AFL", "AFL TH mask");
 rename("Thresholded AFL");

 *// Generate label map using MorphoLibJ. Minimum object volume = 10 voxels*
 selectImage("AFL TH mask");
 run("Size Opening 2D/3D", "min=10");
 run("Connected Components Labeling", "connectivity=6 type=[16 bits]");
 rename("AFL labels");
 run("16-bit");

 *// 2.4. Obtain measurements for all objects in AFL label map*
 *// Initialise and reset 3D ROI manager*
 run("3D Manager");
 Ext.Manager3D_Reset();

 *// Add AFL label map to 3D ROI manager and apply all ROIs to smoothed AFL stack*
 selectImage("AFL labels");
 Ext.Manager3D_AddImage;
 selectImage("Smoothed AFL");
 Ext.Manager3D_SelectAll;
 Ext.Manager3D_Quantif;
 Ext.Manager3D_Measure;
 selectWindow("QuantifTable");
 saveAs("Text", OutputDir+"All AFL_1"+imageTitle);
 run("Close");
 selectWindow("MeasureTable");
 saveAs("Text", OutputDir+"All_ AFL_2"+imageTitle);
 run("Close");

 *// Close all results windows and reset 3D ROI manager*
 Ext.Manager3D_CloseResult("M");
 Ext.Manager3D_Reset();
 run("Clear Results");


selectWindow(imageTitle1);
 run("Duplicate...", "duplicate channels=2");
 rename("Raw Iba-1");
 run("Median 3D...", "x=1 y=1 z=0.3");
 rename("Smoothed Iba-1");

*//run("Merge Channels...", "c1=[Thresholded AFL] c2=[Raw Iba-1] create keep");*
*//	saveAs("Tiff", OutputDir+"Thresholded stacks/TH_"+imageTitle);*
*//	rename("Thresholded stack");*

*//	run("CLIJ2 Macro Extensions", "cl_device=[Quadro P3200]");*
 run("CLIJ2 Macro Extensions", "cl_device=");
 Ext.CLIJ2_clear();

*// 3. Processing of Iba-1 channel (channel #2 in 2-channel stacks)*
 *// 3.1 Generating a microglia soma label map in GPU*
 *// Send Iba-1 channel to GPU*
 selectWindow(imageTitle);
 Stack.setChannel(2);
 Ext.CLIJ2_pushCurrentZStack(imageTitle1;

 *// Pre-processing*
 *// Smooth*
 Ext.CLIJ2_median3DSphere(imageTitle1, Iba1_Smoothed, 3.0, 3.0, 1.0);
 *// Subtract gaussian background*
 Ext.CLIJx_subtractBackground3D(Iba1_Smoothed, BGsub_Iba1, 40, 40, 16.6);

 *// Create a microglia soma mask*
 *// Enhance intensity of large objects using a 3D greyscale opening filter*
 Ext.CLIJ2_greyscaleOpeningSphere(Iba1_Smoothed1, Opening, 15.0, 15.0, 5.0);
 *// Generate a binary mask using MaxEntropy thresholding*
 Ext.CLIJ2_automaticThreshold(Opening, TH, "MaxEntropy");
 *// Mask processing: fill holes and apply opening and closing filters to remove any leftover processes*
 Ext.CLIJx_binaryFillHolesSliceBySlice(TH, Fillholes);
 number_of_erotions_and_dilations = 10.0;
 Ext.CLIJ2_openingDiamond(Fillholes, Opening, number_of_erotions_and_dilations);
 number_of_dilations_and_erotions = 10.0;
 Ext.CLIJ2_closingDiamond(Opening, Closing, number_of_dilations_and_erotions);

 *// Generate label map from microglia soma*
 Ext.CLIJ2_connectedComponentsLabelingDiamond(Closing, SomaLbls);
 Ext.CLIJ2_excludeLabelsOutsideSizeRange(SomaLbls, SomaLbls_1, 50000, 1000000);
 Ext.CLIJ2_dilateLabels(SomaLbls_1, SomaLbls_2, 1.5);

 *// Edit label map properties for visualisation*
 Ext.CLIJ2_detectLabelEdges(SomaLbls_2, labelmap_edges);
 *// make borders a bit wider*
 Ext.CLIJ2_dilateBox(labelmap_edges, labelmap_edges_dilated);
 *// bring label numbers (colours) to the label edge image*
 Ext.CLIJ2_mask(SomaLbls_2, labelmap_edges_dilated, labelmap_outlines);
 *// visualize labeled image*

 *// 3.2 Visualisation and analysis of microglia soma labels in Image J*
 *// Soma label outlines image*
 Ext.CLIJ2_pull(labelmap_outlines);
 run("glasbey_on_dark");
 resetMinAndMax;
 rename("Soma outlines");
 run("16-bit");

 *// Bring label map from GPU to ImageJ*
 Ext.CLIJ2_pull(SomaLbls_2);
 run("glasbey on dark");
 resetMinAndMax;
 rename("Soma labels");
 run("16-bit");

 *// Get number of microglia soma and position and add numerical labels to map*
 run("Clear Results");
 *// measure maximum intensity in the label image which equals the number of labeled objects*
 Ext.CLIJ2_getMaximumOfAllPixels(SomaLbls_2, number_of_labels);
 *// determine mass center (and other statistics)*
 Ext.CLIJ2_statisticsOfLabelledPixels(imageTitle, SomaLbls_2);

 *// configure font for drawing text*
 labelFontSize = 24;
 labelFontColor = "yellow";
 style = "bold italic";
 setFont("SansSerif", labelFontSize, style);
 setColor(labelFontColor);

*// 4. Analysis of autofluorescent granules within microglia.*
*// 4.0 Finish stack processing if no microglia soma were identified in step 3 or continue on.*
 **if** (number_of_labels==0) {
 print("No microglia soma found in "+imageTitle+". Processing done.");
 close("*");
 }
 **else** {
 *// 4.1. Generate and save stack with microglia soma numbers and outlines for easy identification of cells that meet exclusion criteria*
 selectWindow("Smoothed Iba-1");
 run("Duplicate...", "duplicate");
 rename("Outlines Iba-1");

 *// Draw the label numbers as overlays. The x,y coordinates are retrieved from the label statistics in the results table.*
 **for** (i = 0; i < number_of_labels; i++) {
 x = getResult("MASS_CENTER_X", i);
 y = getResult("MASS_CENTER_Y", i);
 Overlay.drawString(i+1, x - labelFontSize/2, y + labelFontSize/2);
 }
 *// Create and save stack with smoothed Iba-1 and soma label numbers. Note that this step can be omitted unless the microglia maskign is run separately.*
 run("Merge Channels...", "c1=[Outlines Iba-1] c2=[Soma outlines] create keep");
 Overlay.show();
 saveAs("Tiff", OutputDir+"Soma maps/Soma numbers_"+imageTitle);
 close();
 run("Clear Results");

 *// 4.2 Generate a stack with smoothed Iba-1, thresholded AFL and corresponding label numbers and save for any further analysis required*
 run("Merge Channels...", "c1=[Smoothed Iba-1] c2=[Thresholded AFL] c3=[Soma labels] c4=[AFL labels] create keep");
 saveAs("Tiff", OutputDir+"Labelled stacks/Lbl_"+imageTitle);

 *// 4.3 Analyse AFL and Iba-1 overlap using Biovoxxel 3D Box Object Inspector*
 *// Split channels from previous stack*
 rename("Labels");
 run("Split Channels");
 selectImage("C1-Labels");
 rename("Iba-1");
 selectImage("C2-Labels");
 rename("AFL");
 selectImage("C3-Labels");
 rename("Microglia");
 selectImage("C4-Labels");
 rename("Aggregates");

 *// Apply Object Inspector. Primary objects = microglia soma, secondary objects = AFL aggregates*
 run("Object Inspector (2D/3D)", "primary_imageplus=Microglia secondary_imageplus=Aggregates original_1_title=Iba-1 original_2_title=AFL"+ " primary_volume_range=0-Infinity"+" primary_mmer_range=0.00-1.00 secondary_volume_range=10.0-Infinity secondary_mmer_range=0.00-1.00"+" exclude_primary_objects_on_edges=false pad_stack_tops=false display_results_tables=true display_analyzed_label_maps=true show_count_map=false");
 *// Save primary results table containing soma volume, Iba-1 intensity and number of AFL aggregates for each microglia*
 selectWindow("Primary_Results");
 saveAs("Text", OutputDir+"Cells/Microglia_"+imageTitle);
 run("Close");
 *// Save secondary results table detailing the microglia in which each AFL aggregate is located, plus volume, intensity, etc for each AFL aggregate*
 selectWindow("Secondary_Results");
 saveAs("Text", OutputDir+"AFL/Aggregates_"+imageTitle);
 run("Close");

 *// Save results stack for manual revision*
 run("Merge Channels...", "c1=[final_Aggregates] c2=[final_Microglia] c3=[Smoothed Iba-1] c4=[Soma outlines] create keep");
 saveAs("Tiff", OutputDir+"Object inspector/Numbers_"+imageTitle);

 print(imageTitle+" finished processing.");
}

*// Close all open images*
close("*");
