# Supplementary material for: Establishing a Biophotonic Signature of Brain Ageing
Source: Aging Cell. 2026 Nov 1;25(11):e70790. doi: 10.1111/acel.70790 (PMC13724186; doi:10.1111/acel.70790)
Supplement: Supplementary file 2 [file ACEL-25-e70790-s002.docx]

**Supplementary Table 1.** Taqman primer details.

| **Target Gene** | **NCBI Reference Sequence** | **Taqman Assay ID** | **Product Size** |
| --- | --- | --- | --- |
| *Gapdh* | NM_001289726.1 | Mm99999915_g1 | 107 |
| *Hprt* | NM_013556.2 | Mm03024075_m1 | 131 |
| *Abca7* | NM_013850.1 | Mm00497010_m1 | 68 |
| *Abcg1* | NM_009593.2 | Mm00437390_m1 | 65 |
| *Acat1* | NM_144784.3 | Mm00507463_m1 | 95 |
| *Aif1* | NM_019467.2 | Mm00479862_g1 | 82 |
| *App* | NM_001198823.1 | Mm01344172_m1 | 111 |
| *Bace1* | NM_001145947.1 | Mm00478664_m1 | 124 |
| *Bace2* | NM_019517.4 | Mm00517138_m1 | 64 |
| *Ch25h* | NM_009890.1 | Mm00515486_s1 | 126 |
| *Hexb* | NM_010422.2 | Mm01282432_m1 | 80 |
| *Itgam* | NM_001082960.1 | Mm00434455_m1 | 69 |
| *Lpl* | NM_008509.2 | Mm00434764_m1 | 61 |
| *Mapt* | NM_001038609.2 | Mm00521988_m1 | 58 |
| *Mhc-II/Cd74* | NM_001042605.1 | Mm00658576_m1 | 118 |
| *mTor* | NM_020009.2 | Mm00444968_m1 | 65 |
| *Nf-kb1* | NM_008689.2 | Mm00476361_m1 | 70 |
| *Ptdss1* | NM_008959.3 | Mm00447254_m1 | 74 |
| *Sirt1* | NM_001159589.1 | Mm01168521_m1 | 94 |
| *Sorl1* | NM_011436.3 | Mm01169526_m1 | 78 |
| *Tlr4* | NM_021297.2 | Mm00445273_m1 | 87 |

**Supplementary Table 2.** Age-associated plasma lipids without significant associations with brain spectral ratios (fold change >1.5, FDR <0.1).

| **Lipid** | **Unstd. Coefficients** | | **t** | **Sig.** |
| --- | --- | --- | --- | --- |
|  | Beta | Std. Error |  |  |
| PE 36:5 | -0.13 | 0.065 | -2.02 | 0.057 |
| PE 38:5 | -0.16 | 0.077 | -2.007 | 0.058 |
| AcylCarnitine 14:0 | 0.26 | 0.13 | 2.01 | 0.058 |
| PC 36:6 | -0.20 | 0.099 | -1.99 | 0.06 |
| PE(P-16:0_20:5) | -0.34 | 0.18 | -1.97 | 0.063 |
| AcylCarnitine 13:0 | 0.23 | 0.13 | 1.83 | 0.083 |
| CE 20:5 | -0.24 | 0.13 | -1.81 | 0.086 |
| PI 36:1 | 0.27 | 0.15 | 1.76 | 0.093 |
| AcylCarnitine 12:0 | 0.20 | 0.12 | 1.63 | 0.12 |
| PI 40:5 | -0.11 | 0.090 | -1.27 | 0.22 |

**Supplementary Table 3.** Simple linear regression with brain spectra ratio and the two candidate plasma lipid intensities in the validation cohort with the tumour-bearing animal excluded (grey) and included (white).

| **Lipid** | **Unstd. Coefficients** | | **t** | **Sig.** |
| --- | --- | --- | --- | --- |
|  | Beta | Std. Error |  |  |
| SM 32:2 | -0.98 | 0.27 | -3.64 | 0.005 |
| PE 38:6 | -0.42 | 0.59 | -0.72 | 0.49 |
| SM 32:2 | -0.98 | 0.26 | -3.82 | 0.003 |
| PE 38:6 | -0.42 | 0.56 | -0.75 | 0.47 |

**Supplementary Table 4.** Simple linear regression of the remaining lipids from the 9 original cohort lipids (non-significant after Bonferroni correction) assessed in the validation cohort (*p* < 0.05). PE 40:6 and AcylCarnitine 18:2 were not detected in this validation cohort.

| **Lipid** | **Unstd. Coefficients** | | **t** | **Sig.** |
| --- | --- | --- | --- | --- |
|  | Beta | Std. Error |  |  |
| AcylCarnitine 16:0 | 0.55 | 0.20 | 2.70 | 0.022 |
| PE 36:4 | -0.70 | 0.27 | -2.63 | 0.025 |
| PE 38:4 | -1.23 | 0.55 | -2.23 | 0.050 |
| PC 36:5 | -0.56 | 0.45 | -1.26 | 0.24 |
| PE(18:0_20:5) | -0.52 | 0.48 | -1.09 | 0.30 |
| PE 40:7 | 0.38 | 0.68 | 0.55 | 0.59 |
| PE 40:5 | 0.16 | 0.54 | 0.29 | 0.78 |
