# Supplementary material for: Establishing a Biophotonic Signature of Brain Ageing
Source: Aging Cell. 2026 Nov 1;25(11):e70790. doi: 10.1111/acel.70790 (PMC13724186; doi:10.1111/acel.70790)
Supplement: Supplementary file 3 [file ACEL-25-e70790-s003.docx]

**Supplementary Figure 1**

**Supplementary Fig. 1. A schematic representation of time-correlated single photon counting (TCSPC)-based scanning fluorescence lifetime imaging.** A pulsed 485 nm laser is focused on a region-of-interest around the autofluorescent aggregate, which is scanned and dedicated using timing electronics and a single point detector. Different emission windows are acquired by manually changing a cube containing a mirror and filters. DBS: dichroic beamsplitter; TCSPC: time-correlated single photon counting. Figure created with BioRender.com; Toronto, Canada.

**Supplementary Figure 2**

**Supplementary Fig. 2. Aging leads to accumulation of autofluorescent material in the hippocampus and ARC.** A-D) compared to young, aged animals have more autofluorescent material detected through 488 nm excitation in the (A) dentate gyrus (DG), (B) CA1 region of the hippocampus, (C) CA3 region, and (D) arcuate nucleus of the hypothalamus (ARC). E-L) Representative images of autofluorescent material in the (E) young and (F) aged DG, (G) young and (H) aged CA1, (I) young and (J) aged CA3, and (K) young and (L) aged ARC. M, N) autofluorescent material colocalises within Iba1-positive microglia labelled with Alexa 647 in (M) young and (N) aged ARC. Scale bars = 20 µm. * *p* < 0.05, *** *p* < 0.001. Data are mean ± SEM. n = 4-5.

**Supplementary Figure 3**

**Supplementary Fig. 3. Microglia autofluorescence volume is associated with branching complexity in the ARC but not the hippocampus.** The number of microglia branches are not associated with the volume of microglial autofluorescence in the A) DG, B) CA1, or C) CA3. D) In the ARC, there is a significant, moderate relationship between microglial branching and volume of microglial autofluorescence (*p* = 0.027). Data points represent the mean values of individual animals (*n* = 10). Blue circles = young, white triangles = aged.

**Supplementary Figure 4**

**Supplementary Fig. 4.** Second derivative analysis of the spectral emission showing all identified peaks including those with <90% presence in all animals in A) original cohort and B) validation cohort. C) A comparison of intensities at all identified peaks in the original cohort. Like the selective analysis for the original cohort, there was still a significant interaction between age and size for normalised intensity across all peaks identified in the second derivative spectrum (F_(34, 555)_ = 1.89, *p* = 0.002). At 507 nm, young animals still had significantly higher intensities than aged, small (*p* = 0.026) and aged, large (*p* < 0.001) autofluorescence. Similarly at this peak, aged, small autofluorescent aggregates were still more intense than aged, large (*p* = 0.004). Young aggregates also had a significant higher intensity than aged, large autofluorescent aggregates at 546 nm (*p* = 0.004) and 731 nm (*p* 0.047). D) A comparison of intensities at all identified peaks in the validation cohort. Like the selective analysis for the validation cohort, there was still a significant interaction between age and size for normalised intensity across all peaks identified in the second derivative spectrum (F_(34, 269)_ = 2.12, *p* < 0.001). Young aggregates had a significantly higher intensity than aged, large aggregates at 507 nm (*p* = 0.006) and 524 nm (*p* = 0.039). At 608 nm, young aggregates had a significantly lower intensity than aged, small (*p* = 0.028) and aged, large (*p* = 0.013) aggregates but there was no significant difference at 731 nm in this second cohort. **p* < 0.05. ***p* < 0.01. *** *p* < 0.001. Data are mean ± SEM. n = 10-12 per group and 6 per group in each cohort, respectively.

**Supplementary Figure 5**

**Supplementary Fig. 5.** A similar spectral pattern is observed with age when including the tumour-bearing animal (indicated by the red data point). A) Mean ratio of intensities between the emission at 515-555 nm and 630-670 nm. B) Comparison of intensities at each identified peak. Young aggregates had a significantly higher intensity than aged, large at 507 nm (*p* = 0.012) and 524 nm (*p* = 0.043). Aged, large aggregates had a significantly higher intensity than young (*p* = 0.009) and aged, small (*p* = 0.012) aggregates at 608 nm and 731 nm, respectively. **p* < 0.05. ***p* < 0.01. Data are mean ± SEM. n = 6-7 per group.
